# Extensions of different type parameterized inequalities for generalized $(m,h)$-preinvex mappings via *k*-fractional integrals

**DOI:** 10.1186/s13660-018-1639-5

**Published:** 2018-02-21

**Authors:** Yao Zhang, Ting-Song Du, Hao Wang, Yan-Jun Shen, Artion Kashuri

**Affiliations:** 1 Department of Mathematics, College of Science, China Three Gorges University, Yichang, China; 2 Three Gorges Mathematical Research Center, China Three Gorges University, Yichang, China; 3 Hubei Provincial Collaborative Innovation Center for New Energy Microgrid, China Three Gorges University, Yichang, China; 4 Department of Mathematics, Faculty of Technical Science, University “Ismail Qemali”, Vlora, Albania

**Keywords:** 26A33, 26A51, 26D07, 26D20, 41A55, Hermite–Hadamard’s inequality, Simpson’s inequality, Generalized $(m,h)$-preinvex functions, *k*-fractional integrals

## Abstract

The authors discover a general *k*-fractional integral identity with multi-parameters for twice differentiable functions. By using this integral equation, the authors derive some new bounds on Hermite–Hadamard’s and Simpson’s inequalities for generalized $(m,h)$-preinvex functions through *k*-fractional integrals. By taking the special parameter values for various suitable choices of function *h*, some interesting results are also obtained.

## Introduction

The subsequent inequalities are notable in the literature as Hermite–Hadamard’s inequality and Simpson’s inequality, respectively.

### Theorem 1.1

*Suppose that* $f:I\subseteq \mathbb{R}\rightarrow \mathbb{R}$ *is a convex function defined on the interval* *I* *of real numbers and* $a,b\in I$ *along with* $a< b$. *The following double inequality holds*:

$$ f \biggl(\frac{a+b}{2} \biggr)\leq \frac{1}{b-a} \int ^{b}_{a} f(x)\,\mathrm{d}x\leq \frac{f(a)+f(b)}{2}. $$

### Theorem 1.2

*Assume that* $f:[a,b]\rightarrow \mathbb{R}$ *is a four times continuously differentiable mapping on* $(a,b)$ *and* $\Vert f^{(4)} \Vert _{\infty }=\mathrm{sup}_{x\in (a,b)} \vert f^{(4)}(x) \vert <\infty $. *Then the following inequality holds*:

$$ \biggl\vert \frac{1}{3} \biggl[ \frac{f(a)+f(b)}{2}+2f \biggl(\frac{a+b}{2} \biggr) \biggr]-\frac{1}{b-a} \int ^{b}_{a} f(x)\,\mathrm{d}x \biggr\vert \leq \frac{1}{2880} \bigl\Vert f^{(4)} \bigr\Vert _{\infty }(b-a)^{4}. $$

Hermite–Hadamard’s inequalities and Simpson’s inequalities have remained an area of great interest owing to their extensive applications in mathematics and other sciences. Many researchers generalized these inequalities. For recent results, for example, see [1–8] and the references mentioned in these papers.

In 2013, Sarikaya et al. established the subsequent interesting Hermite–Hadamard’s inequalities by utilizing Riemann–Liouville fractional integrals.

### Theorem 1.3

([9])

*Let* $f:[a,b]\rightarrow \mathbb{R}$ *be a positive function along with* $0\leq a< b$, *and let* $f\in L^{1}[a,b]$. *Suppose that* *f* *is a convex function on* $[a,b]$, *then the following inequalities for fractional integrals hold*:

1.1 $$ f \biggl(\frac{a+b}{2} \biggr) \leq \frac{\Gamma (\mu +1)}{2(b-a)^{\mu }}\bigl[J^{\mu }_{a^{+}}f(b)+J^{\mu }_{b^{-}}f(a) \bigr]\leq \frac{f(a)+f(b)}{2}, $$

*where the symbols* $J^{\mu }_{a^{+}} f$ *and* $J^{\mu }_{b^{-}} f$ *denote respectively the left*-*sided and right*-*sided Riemann–Liouville fractional integrals of order* $\mu >0$ *defined by*

$$ J^{\mu }_{a^{+}}f(x) = \frac{1}{\Gamma (\mu )} \int ^{x}_{a}(x-t)^{\mu -1}f(t)\,\mathrm{d}t, \quad a< x, $$

*and*

$$ J^{\mu }_{b^{-}}f(x) = \frac{1}{\Gamma (\mu )} \int ^{b}_{x}(t-x)^{\mu -1}f(t)\,\mathrm{d}t, \quad x< b. $$

*Here*, $\Gamma (\mu )$ *is the gamma function and its definition is* $\Gamma (\mu )=\int _{0}^{\infty }e^{-t}t^{\mu -1}\,\mathrm{d}t$. *It is to be noted that* $J^{0}_{a^{+}}f(x)=J^{0}_{b^{-}}f(x)=f(x)$.

In the case of $\mu =1$, the fractional integral recaptures the classical integral.

Because of the extensive application of Riemann–Liouville fractional integrals, some authors extended their studies to fractional Hermite–Hadamard’s inequalities via mappings of different classes. For example, refer to [10–12] for convex mappings, to [13] for *s*-convex mappings, to [14] for $(s,m)$-convex mappings, to [15] for *s*-Godunova–Levin mappings, to [16] for harmonically convex mappings, to [17] for preinvex mappings, to [18] for MT_*m*_-preinvex mappings, to [19] for *h*-convex mappings, to [20] for *r*-convex mappings, and see the references cited therein.

In 2012, Mubeen and Habibullah introduced the following class of fractional integrals.

### Definition 1.1

([21])

Let $f\in L^{1}[a,b]$, then the Riemann–Liouville *k*-fractional integrals ${}_{k}J^{\mu }_{a^{+}}f(x)$ and ${}_{k}J^{\mu }_{b^{-}}f(x)$ of order $\mu >0$ are given as

$$ {}_{k}J^{\mu }_{a^{+}}f(x) = \frac{1}{k\Gamma _{k}(\mu )} \int ^{x}_{a}(x-t)^{\frac{\mu }{k}-1}f(t)\,\mathrm{d}t \quad (0\leq a< x< b) $$

and

$$ {} _{k}J^{\mu }_{b^{-}}f(x) = \frac{1}{k\Gamma _{k}(\mu )} \int ^{b}_{x}(t-x)^{\frac{\mu }{k}-1}f(t)\,\mathrm{d}t\quad (0\leq a< x< b), $$

respectively, where $k>0$ and $\Gamma _{k}(\mu )$ is the *k*-gamma function defined by $\Gamma _{k}(\mu )=\int ^{\infty }_{0} t^{\mu -1} e^{-\frac{t^{k}}{k}}\,\mathrm{d}t$. Furthermore, $\Gamma _{k}(\mu +k)=\mu \Gamma _{k}(\mu )$ and ${}_{k}J^{0}_{a^{+}}f(x)={}_{k}J^{0}_{b^{-}}f(x)=f(x)$.

The concept of Riemann–Liouville *k*-fractional integral is an important generalization of Riemann–Liouville fractional integrals. We would like to stress here that for $k\neq 1$ the properties of Riemann–Liouville *k*-fractional integrals are very dissimilar to those of classical Riemann–Liouville fractional integrals. Due to this, the Riemann–Liouville *k*-fractional integrals have aroused many researchers’ interest. Properties and estimations for the integral inequality related to this operator can be sought out in [22–27] and the references cited therein.

The main purpose of the current paper is to establish some new bounds on Hermite–Hadamard’s and Simpson’s inequalities for mappings whose absolute values of second derivatives are generalized $(m,h)$-preinvex. To do this, the authors derive a general *k*-fractional integral identity along with multi parameters for twice differentiable mappings. By using this integral identity, the authors derive some new inequalities of Simpson and Hermite–Hadamard type for these mappings.

To end this section, we restate some special functions and definitions as follows.

Let us consider the following special functions:

1. The beta function:
  $$ \begin{aligned} \beta (x,y)=\frac{\Gamma (x)\Gamma (y)}{\Gamma (x+y)}= \int ^{1}_{0}t^{x-1}(1-t)^{y-1}\,\mathrm{d}t, \quad x,y>0; \end{aligned} $$
2. The incomplete beta function:
  $$ \begin{aligned} \beta (a;x,y)= \int ^{a}_{0}t^{x-1}(1-t)^{y-1}\,\mathrm{d}t, \quad 0< a< 1,x,y>0; \end{aligned} $$
3. The hypergeometric function:
  $$ {}_{2}F_{1}(a,b;c;z)= \frac{1}{\beta (b,c-b)} \int ^{1}_{0} t^{b-1}(1-t)^{c-b-1}(1-zt)^{-a}\,\mathrm{d}t,\quad c>b>0,\vert z \vert < 1. $$

### Definition 1.2

([28])

A function $f : [0,\infty )\rightarrow \mathbb{R}$ is named *s*-convex in the second sense along with $s\in (0,1]$ if

$$ \begin{aligned} f(\alpha x +\beta y)\leq \alpha ^{s} f(x)+ \beta ^{s} f(y) \end{aligned} $$

holds for all $x,y\in [0,\infty )$ and $\alpha ,\beta \geq 0$ along with $\alpha +\beta =1$.

### Definition 1.3

([29])

A function $f :A \subseteq \mathbb{R}\rightarrow \mathbb{R}$ is called *s*-Godunova–Levin function of the second kind along with $s\in [0,1]$ if

$$ \begin{aligned} f \bigl(tx+(1-t)y \bigr)\leq \frac{f(x)}{t^{s}}+ \frac{f(y)}{(1-t)^{s}} \end{aligned} $$

holds for all $x,y\in A$ and $t\in (0,1)$.

### Definition 1.4

([30])

A function $f:A\subseteq \mathbb{R}\rightarrow \mathbb{R}$ is named $tgs$-convex on *A* if *f* is non-negative and

$$ f \bigl(tx+ (1-t)y \bigr)\leq t(1-t)\bigl[f(x)+f(y) \bigr] $$

holds for all $x, y \in A $ and $t\in (0,1)$.

### Definition 1.5

([31])

A function $f: A\subseteq \mathbb{R}\rightarrow \mathbb{R}$ is called *MT*-convex if *f* is non-negative and

$$ f \bigl(tx+(1-t)y \bigr)\leq \frac{\sqrt{t}}{2\sqrt{1-t}}f(x)+\frac{\sqrt{1-t}}{2\sqrt{t}}f(y) $$

holds for all $x,y\in A$ and $t\in (0, 1)$.

### Definition 1.6

([32])

A set $A\subseteq \mathbb{R}^{n}$ is called *m*-invex with respect to the mapping $\eta :A \times A\times (0, 1]\rightarrow \mathbb{R}^{n}$ for some fixed $m\in (0, 1]$ if $mx+\lambda \eta (y, x, m) \in A$ holds for all $x, y \in A$ and $\lambda \in [0, 1]$.

### Definition 1.7

([33])

Let $A \subseteq \mathbb{R}$ be an open *m*-invex subset with respect to $\eta : A \times A \times (0,1]\rightarrow \mathbb{R}$, and let $h_{1}, h_{2} :[0,1]\rightarrow \mathbb{R}_{0}$. A function $f:A\rightarrow \mathbb{R}$ is said to be generalized $(m, h_{1}, h_{2})$-preinvex if

1.2 $$ f \bigl(m x+ t\eta (y, x, m) \bigr)\leq mh_{1}(t) f(x)+ h_{2}(t) f(y) $$

is valid for all $x, y \in A$ and $t \in [0, 1]$. If inequality (1.2) reverses, then *f* is said to be generalized $(m, h_{1}, h_{2})$-preincave on *A*.

Clearly, if we take $h_{1}(t)=h(1-t)$, $h_{2}(t)=h(t)$ in Definition 1.7, then *f* becomes generalized $(m,h)$-preinvex functions as follows.

### Definition 1.8

Let $A\subseteq \mathbb{R}$ be an open *m*-invex subset with respect to $\eta : A\times A\times (0,1] \rightarrow \mathbb{R}$, and let $h :[0,1]\rightarrow \mathbb{R}_{0}$. A function $f:A\rightarrow \mathbb{R}$ is called generalized $(m,h)$-preinvex if

1.3 $$ f \bigl(mx+t\eta (y,x,m) \bigr)\leq h(t)f(y)+mh(1-t)f(x) $$

is valid for all $x, y \in A$ and $t \in [0,1]$.

### Remark 1.1

Let us discuss some special cases of Definition 1.8 as follows:

(i) choosing $h(t)=1$, we obtain the definition of generalized $(m,P)$-preinvex functions;
(ii) choosing $h(t)=t^{s}$ for $s\in (0,1]$, we obtain the definition of generalized $(m,s)$-Breckner-preinvex functions;
(iii) choosing $h(t)=t^{-s}$ for $s\in (0,1)$, we obtain the definition of generalized $(m,s)$-Godunova–Levin–Dragomir-preinvex functions;
(iv) choosing $h(t)=t(1-t)$, we obtain the definition of generalized $(m, tgs)$-preinvex functions;
(v) choosing $h(t)=\frac{\sqrt{t}}{2\sqrt{1-t}}$, we obtain the definition of generalized *m*-*MT*-preinvex functions.

It is worth mentioning here that, as far as we know, all the special cases considered above are new in the literature.

## Main results

In order to derive our main results, we need the subsequent identity.

### Lemma 2.1

*Let* $A\subseteq \mathbb{R}$ *be an open* *m*-*invex subset with respect to* $\eta : A\times A\times (0,1] \rightarrow \mathbb{R}\setminus \{0\}$ *for some fixed* $m \in (0,1]$, *and let* $a, b \in A$, $a< b$ *with* $\eta (b,a,m)>0$. *Assume that* $f:A\rightarrow \mathbb{R}$ *is a twice differentiable function on* *A* *such that* $f''$ *is integrable on* $[ma,ma+\eta (b,a,m) ]$. *Then the following identity for Riemann–Liouville* *k*-*fractional integrals along with* $x \in [a, b]$, $\lambda \in [0, 1]$, $\mu > 0$, *and* $k>0$ *exists*:

2.1 $$\begin{aligned} &I_{f, \eta }(\mu ,k;x,\lambda ,m,a,b) \\ &\quad =\frac{\eta ^{\frac{\mu }{k}+2}(x,a,m)}{(\frac{\mu }{k}+1)\eta (b,a,m)} \int ^{1}_{0}t \biggl[ \biggl(\frac{\mu }{k}+1 \biggr)\lambda -t^{\frac{\mu }{k}} \biggr] f'' \bigl(ma+t \eta (x,a,m) \bigr)\,\mathrm{d}t \\ &\quad \quad {} +\frac{(-1)^{\frac{\mu }{k}+2}\eta ^{\frac{\mu }{k}+2}(x,b,m)}{(\frac{\mu }{k}+1)\eta (b,a,m)} \int ^{1}_{0}t \biggl[ \biggl(\frac{\mu }{k}+1 \biggr)\lambda -t^{\frac{\mu }{k}} \biggr] f'' \bigl(mb+t \eta (x,b,m) \bigr)\,\mathrm{d}t, \end{aligned}$$

*where*

$$\begin{aligned} &I_{f, \eta }(\mu ,k;x,\lambda ,m,a,b) \\ &\quad:=\frac{1-\lambda }{\eta (b,a,m)} \bigl[\eta ^{\frac{\mu }{k}}(x,a,m)f \bigl(ma+\eta (x,a,m) \bigr) \\ &\quad\quad {} +(-1)^{\frac{\mu }{k}}\eta ^{\frac{\mu }{k}}(x,b,m)f \bigl(mb+\eta (x,b,m) \bigr) \bigr] \\ &\quad\quad {} +\frac{\lambda }{\eta (b,a,m)} \bigl[\eta ^{\frac{\mu }{k}}(x,a,m)f(ma) +(-1)^{\frac{\mu }{k}}\eta ^{\frac{\mu }{k}}(x,b,m)f(mb) \bigr] \\ &\quad\quad {} +\frac{\frac{1}{\frac{\mu }{k}+1}-\lambda }{\eta (b,a,m)} \bigl[(-1)^{\frac{\mu }{k}+1}\eta ^{\frac{\mu }{k}+1}(x,b,m)f' \bigl(mb+\eta (x,b,m) \bigr) \\ &\quad\quad {} -\eta ^{\frac{\mu }{k}+1}(x,a,m)f' \bigl(ma+\eta (x,a,m) \bigr) \bigr] \\ &\quad \quad -\frac{\Gamma _{k}(\mu +k)}{\eta (b,a,m)} \bigl[{}_{k}J^{\mu }_{(ma+\eta (x,a,m))^{-}} f(ma)+{}_{k}J^{\mu }_{(mb+\eta (x,b,m))^{+}}f(mb) \bigr] \end{aligned}$$

*and* $\Gamma _{k}$ *is the* *k*-*gamma function*.

### Proof

By integration by parts and replacing the variable, we can state

2.2 $$\begin{aligned} &\int ^{1}_{0}t \biggl[ \biggl(\frac{\mu }{k}+1 \biggr)\lambda -t^{\frac{\mu }{k}} \biggr]f'' \bigl(ma+t \eta (x,a,m) \bigr)\,\mathrm{d}t \\ &\quad =t \biggl[ \biggl(\frac{\mu }{k}+1 \biggr)\lambda -t^{\frac{\mu }{k}} \biggr] \frac{f' (ma+t\eta (x,a,m) )}{\eta (x,a,m)} \bigg| _{0}^{1} \\ &\quad \quad {} -\frac{\frac{\mu }{k}+1}{\eta (x,a,m)} \int ^{1}_{0} \bigl(\lambda -t^{\frac{\mu }{k}} \bigr)f' \bigl(ma+t\eta (x,a,m) \bigr)\,\mathrm{d}t \\ &\quad = \biggl[ \biggl(\frac{\mu }{k}+1 \biggr)\lambda -1 \biggr] \frac{f' (ma+\eta (x,a,m) )}{\eta (x,a,m)} \\ &\quad \quad {} -\frac{\frac{\mu }{k}+1}{\eta (x,a,m)} \biggl[ \bigl(\lambda -t^{\frac{\mu }{k}} \bigr) \frac{f (ma+t\eta (x,a,m) )}{\eta (x,a,m)} \bigg| _{0}^{1} \\ &\quad \quad {} + \int ^{1}_{0}\frac{\mu }{k}t^{\frac{\mu }{k}-1} \frac{f (ma+t\eta (x,a,m) )}{\eta (x,a,m)}\,\mathrm{d}t \biggr] \\ &\quad = \biggl[ \biggl(\frac{\mu }{k}+1 \biggr)\lambda -1 \biggr] \frac{f' (ma+\eta (x,a,m) )}{\eta (x,a,m)} \\ &\quad \quad {} +\frac{\frac{\mu }{k}+1}{\eta ^{2}(x,a,m)} \biggl[(1-\lambda )f \bigl(ma+\eta (x,a,m) \bigr)+ \lambda f(ma) \\ &\quad \quad {} -\frac{\frac{\mu }{k}}{\eta ^{\frac{\mu }{k}}(x,a,m)}\int ^{ma+\eta (x,a,m)}_{ma}(u-ma)^{\frac{\mu }{k}-1}f(u)\,\mathrm{d}u \biggr] \\ &\quad = \biggl[ \biggl(\frac{\mu }{k}+1 \biggr)\lambda -1 \biggr]\frac{f' (ma+\eta (x,a,m) )}{\eta (x,a,m)} \\ &\quad \quad {} +\frac{\frac{\mu }{k}+1}{\eta ^{2}(x,a,m)} \biggl[(1-\lambda )f \bigl(ma+\eta (x,a,m) \bigr)+ \lambda f(ma) \\ &\quad \quad {} -\frac{\Gamma _{k}(\mu +k)}{\eta ^{\frac{\mu }{k}}(x,a,m)}{}_{k}J^{\mu }_{(ma+\eta (x,a,m))^{-}}f(ma)\biggr]. \end{aligned}$$

Similarly, we get

2.3 $$\begin{aligned} &\int ^{1}_{0}t \biggl[ \biggl(\frac{\mu }{k}+1 \biggr)\lambda -t^{\frac{\mu }{k}} \biggr]f'' \bigl(mb+t \eta (x,b,m) \bigr)\,\mathrm{d}t \\ &\quad = \biggl[ \biggl(\frac{\mu }{k}+1 \biggr)\lambda -1 \biggr] \frac{f' (mb+\eta (x,b,m) )}{\eta (x,b,m)} \\ &\quad \quad {} +\frac{\frac{\mu }{k}+1}{\eta ^{2}(x,b,m)} \biggl[(1-\lambda )f \bigl(mb+\eta (x,b,m) \bigr)+ \lambda f(mb) \\ &\quad \quad {} -\frac{\Gamma _{k}(\mu +k)}{\eta ^{\frac{\mu }{k}}(x,b,m)}{}_{k}J^{\mu }_{(ma+\eta (x,b,m))^{-}}f(mb)\biggr]. \end{aligned}$$

Multiplying both sides of (2.2) and (2.3) by $\frac{\eta ^{\frac{\mu }{k}+2}(x,a,m)}{(\frac{\mu }{k}+1)\eta (b,a,m)}$ and $\frac{(-1)^{\frac{\mu }{k}+2}\eta ^{\frac{\mu }{k}+2}(x,b,m)}{(\frac{\mu }{k}+1)\eta (b,a,m)}$, respectively, and adding the resulting identities together, we obtain the desired result. □

### Remark 2.1

In Lemma 2.1, if we put $k=1$ and $\eta (b,a,m)=b-ma$ along with $m=1$, then we get the following identity:

$$\begin{aligned} &(1-\lambda ) \biggl[\frac{(x-a)^{\mu }+(b-x)^{\mu }}{b-a} \biggr]f(x)+ \lambda \biggl[\frac{(x-a)^{\mu }f(a)+(b-x)^{\mu }f(b)}{b-a} \biggr] \\ &\quad \quad {} + \biggl(\frac{1}{\mu +1}-\lambda \biggr) \biggl[\frac{(b-x)^{\mu +1}-(x-a)^{\mu +1}}{b-a} \biggr]f'(x)-\frac{\Gamma (\mu +1)}{b-a} \bigl[J^{\mu }_{x^{-}}f(a)+J^{\mu }_{x^{+}}f(b) \bigr] \\ &\quad =\frac{(x-a)^{\mu +2}}{(\mu +1)(b-a)} \int ^{1}_{0}t \bigl[ (\mu +1 )\lambda -t^{\mu } \bigr]f'' \bigl(tx+(1-t)a \bigr)\,\mathrm{d}t \\ &\quad \quad {} +\frac{(b-x)^{\mu +2}}{(\mu +1)(b-a)} \int ^{1}_{0}t \bigl[ (\mu +1 )\lambda -t^{\mu } \bigr]f'' \bigl(tx+(1-t)b \bigr)\,\mathrm{d}t, \end{aligned}$$

which is proved by İşcan in [34]. Further, if we put $\mu =1, \lambda =\frac{1}{2}$, and $x=a$ or $x=b$, then the above identity recaptures Lemma 1 in [35].

Using Lemma 2.1, we now state the following theorem.

### Theorem 2.1

*Let* $A\subseteq \mathbb{R}$ *be an open* *m*-*invex subset with respect to* $\eta : A\times A\times (0,1] \rightarrow \mathbb{R}\setminus \{0\}$ *for some fixed* $m \in (0,1]$, *and let* $a, b \in A$, $a< b$ *with* $\eta (b,a,m)>0$. *Assume that* $f:A\rightarrow \mathbb{R}$ *is a twice differentiable function on* *A* *such that* $f''$ *is integrable on* $[ma,ma+\eta (b,a,m) ]$. *If* $\vert f'' \vert ^{q}$ *for* $q\geq 1$ *is a generalized* $(m,h)$-*preinvex function with respect to* *η* *and* $h:[0,1]\rightarrow \mathbb{R}_{0}$, *then the following inequality for* *k*-*fractional integrals with* $x \in [a,b]$, $\lambda \in [0, 1]$, $\mu > 0$, $k>0$ *exists*:

2.4 $$\begin{aligned} & \bigl\vert I_{f,\eta }(\mu,k;x,\lambda ,m,a,b) \bigr\vert \\ &\quad \leq C_{0}^{1-\frac{1}{q}}(k,\mu ,\lambda ) \biggl\{ \biggl\vert \frac{\eta ^{\frac{\mu }{k}+2}(x,a,m)}{(\frac{\mu }{k}+1)\eta (b,a,m)} \biggr\vert \bigl[ \bigl\vert f''(x) \bigr\vert ^{q}C_{1}(k,\mu ,\lambda ;h) \\ &\quad \quad {} +m \bigl\vert f''(a) \bigr\vert ^{q}C_{2}(k,\mu ,\lambda ;h) \bigr]^{\frac{1}{q}} \\ &\quad \quad {} + \biggl\vert \frac{(-1)^{\frac{\mu }{k}+2}\eta ^{\frac{\mu }{k}+2}(x,b,m)}{(\frac{\mu }{k}+1)\eta (b,a,m)} \biggr\vert \bigl[ \bigl\vert f''(x) \bigr\vert ^{q}C_{1}(k, \mu ,\lambda ;h) \\ &\quad \quad {} +m \bigl\vert f''(b) \bigr\vert ^{q}C_{2}(k,\mu ,\lambda ;h) \bigr]^{\frac{1}{q}} \biggr\} , \end{aligned}$$

*where*

2.5 $$\begin{aligned} &C_{0}(k,\mu ,\lambda )= \int ^{1}_{0}t \biggl\vert \biggl( \frac{\mu }{k}+1 \biggr)\lambda -t^{\frac{\mu }{k}} \biggr\vert \,\mathrm{d}t \\ &\hphantom{C_{0}(k,\mu ,\lambda )}=\textstyle\begin{cases} \frac{\frac{\mu }{k}[(\frac{\mu }{k}+1)\lambda ]^{1+\frac{2k}{\mu }}}{\frac{\mu }{k}+2}-\frac{(\frac{\mu }{k}+1)\lambda }{2}+\frac{1}{\frac{\mu }{k}+2},&0\leq \lambda \leq \frac{1}{\frac{\mu }{k}+1}, \\ \frac{(\frac{\mu }{k}+1)\lambda }{2}-\frac{1}{\frac{\mu }{k}+2},&\frac{1}{\frac{\mu }{k}+1}< \lambda \leq 1, \end{cases}\displaystyle \end{aligned}$$

2.6 $$\begin{aligned} & C_{1}(k,\mu ,\lambda ;h)= \int ^{1}_{0}t \biggl\vert \biggl( \frac{\mu }{k}+1 \biggr)\lambda -t^{\frac{\mu }{k}} \biggr\vert h(t)\,\mathrm{d}t,\quad 0\leq \lambda \leq 1, \end{aligned}$$

*and*

2.7 $$ C_{2}(k,\mu ,\lambda ;h)= \int ^{1}_{0}t \biggl\vert \biggl( \frac{\mu }{k}+1 \biggr)\lambda -t^{\frac{\mu }{k}} \biggr\vert h(1-t)\,\mathrm{d}t,\quad 0\leq \lambda \leq 1. $$

### Proof

Applying Lemma 2.1 and the power mean inequality, we have

2.8 $$\begin{aligned} & \bigl\vert I_{f,\eta }(\mu ,k;x,\lambda,m,a,b) \bigr\vert \\ &\quad\leq \biggl\vert \frac{\eta ^{\frac{\mu }{k}+2}(x,a,m)}{(\frac{\mu }{k}+1)\eta (b,a,m)} \biggr\vert \int ^{1}_{0}t \biggl\vert \biggl( \frac{\mu }{k}+1 \biggr)\lambda -t^{\frac{\mu }{k}} \biggr\vert \bigl\vert f'' \bigl(ma+t\eta (x,a,m) \bigr) \bigr\vert \,\mathrm{d}t \\ &\quad\quad {} + \biggl\vert \frac{(-1)^{\frac{\mu }{k}+2}\eta ^{\frac{\mu }{k}+2}(x,b,m)}{(\frac{\mu }{k}+1)\eta (b,a,m)} \biggr\vert \int ^{1}_{0}t \biggl\vert \biggl( \frac{\mu }{k}+1 \biggr)\lambda -t^{\frac{\mu }{k}} \biggr\vert \bigl\vert f'' \bigl(mb+t\eta (x,b,m) \bigr) \bigr\vert \,\mathrm{d}t \\ &\quad\leq \biggl\vert \frac{\eta ^{\frac{\mu }{k}+2}(x,a,m)}{(\frac{\mu }{k}+1)\eta (b,a,m)} \biggr\vert \biggl( \int ^{1}_{0}t \biggl\vert \biggl( \frac{\mu }{k}+1 \biggr)\lambda -t^{\frac{\mu }{k}} \biggr\vert \,\mathrm{d}t \biggr)^{1-\frac{1}{q}} \\ &\quad\quad {} \times \biggl(\int ^{1}_{0}t \biggl\vert \biggl(\frac{\mu }{k}+1 \biggr)\lambda -t^{\frac{\mu }{k}} \biggr\vert \bigl\vert f'' \bigl(ma+t\eta (x,a,m) \bigr) \bigr\vert ^{q}\,\mathrm{d}t \biggr)^{\frac{1}{q}} \\ &\quad\quad {} + \biggl\vert \frac{(-1)^{\frac{\mu }{k}+2}\eta ^{\frac{\mu }{k}+2}(x,b,m)}{(\frac{\mu }{k}+1)\eta (b,a,m)} \biggr\vert \biggl( \int ^{1}_{0}t \biggl\vert \biggl(\frac{\mu }{k}+1 \biggr)\lambda -t^{\frac{\mu }{k}} \biggr\vert \,\mathrm{d}t \biggr)^{1-\frac{1}{q}} \\ &\quad\quad {} \times \biggl(\int ^{1}_{0}t \biggl\vert \biggl( \frac{\mu }{k}+1 \biggr)\lambda -t^{\frac{\mu }{k}} \biggr\vert \bigl\vert f'' \bigl(mb+t\eta (x,b,m) \bigr) \bigr\vert ^{q}\,\mathrm{d}t \biggr)^{\frac{1}{q}} \\ &\quad =C_{0}^{1-\frac{1}{q}}(k,\mu,\lambda ) \\ &\quad \quad {} \times \biggl\{ \biggl\vert \frac{\eta ^{\frac{\mu }{k}+2}(x,a,m)}{(\frac{\mu }{k}+1)\eta (b,a,m)} \biggr\vert \biggl( \int ^{1}_{0}t \biggl\vert \biggl( \frac{\mu }{k}+1 \biggr)\lambda -t^{\frac{\mu }{k}} \biggr\vert \bigl\vert f'' \bigl(ma+t\eta (x,a,m) \bigr) \bigr\vert ^{q}\,\mathrm{d}t \biggr)^{\frac{1}{q}} \\ &\quad \quad {} + \biggl\vert \frac{(-1)^{\frac{\mu }{k}+2}\eta ^{\frac{\mu }{k}+2}(x,b,m)}{(\frac{\mu }{k}+1)\eta (b,a,m)} \biggr\vert \biggl( \int ^{1}_{0}t \biggl\vert \biggl( \frac{\mu }{k}+1 \biggr)\lambda \\ &\quad \quad {} -t^{\frac{\mu }{k}} \biggr\vert \bigl\vert f'' \bigl(mb+t\eta (x,b,m) \bigr) \bigr\vert ^{q}\,\mathrm{d}t \biggr)^{\frac{1}{q}} \biggr\} . \end{aligned}$$

Since $\vert f'' \vert ^{q}$ is generalized $(m,h)$-preinvex on $[ma,ma+\eta (b,a,m) ]$, we get

2.9 $$\begin{aligned} &\int ^{1}_{0}t \biggl\vert \biggl( \frac{\mu }{k}+1 \biggr)\lambda -t^{\frac{\mu }{k}} \biggr\vert \bigl\vert f'' \bigl(ma+t\eta (x,a,m) \bigr) \bigr\vert ^{q}\,\mathrm{d}t \\ &\quad \leq \int ^{1}_{0}t \biggl\vert \biggl( \frac{\mu }{k}+1 \biggr)\lambda -t^{\frac{\mu }{k}} \biggr\vert \bigl[h(t) \bigl\vert f''(x) \bigr\vert ^{q}+mh(1-t) \bigl\vert f''(a) \bigr\vert ^{q} \bigr]\,\mathrm{d}t \\ &\quad = \bigl\vert f''(x) \bigr\vert ^{q}C_{1}(k,\mu ,\lambda ;h)+m \bigl\vert f''(a) \bigr\vert ^{q}C_{2}(k, \mu ,\lambda ;h) \end{aligned}$$

and

2.10 $$\begin{aligned} & \int ^{1}_{0}t \biggl\vert \biggl( \frac{\mu }{k}+1 \biggr)\lambda -t^{\frac{\mu }{k}} \biggr\vert \bigl\vert f'' \bigl(mb+t\eta (x,b,m) \bigr) \bigr\vert ^{q}\,\mathrm{d}t \\ &\quad \leq \int ^{1}_{0}t \biggl\vert \biggl( \frac{\mu }{k}+1 \biggr)\lambda -t^{\frac{\mu }{k}} \biggr\vert \bigl[h(t) \bigl\vert f''(x) \bigr\vert ^{q}+mh(1-t) \bigl\vert f''(b) \bigr\vert ^{q} \bigr]\,\mathrm{d}t \\ &\quad = \bigl\vert f''(x) \bigr\vert ^{q}C_{1}(k,\mu ,\lambda ;h)+m \bigl\vert f''(b) \bigr\vert ^{q}C_{2}(k, \mu ,\lambda ;h), \end{aligned}$$

where $C_{0}(k,\mu ,\lambda )$, $C_{1}(k,\mu ,\lambda ;h)$, and $C_{2}(k,\mu ,\lambda ;h)$ are defined by (2.5)–(2.7), respectively. Hence, if we use (2.9) and (2.10) in (2.8), we can get the desired result. This completes the proof. □

Let us point out some special cases of Theorem 2.1.

**I**. If $h(t)=t^{s}$ in Theorem 2.1, then we have the following results.

### Corollary 2.1

*In Theorem *2.1, *if* $\vert f'' \vert ^{q}$ *for* $q\geq 1$ *is generalized* $(m,s)$-*Breckner*-*preinvex functions*, *then*, *for* $s\in (0,1]$ *and* $m\in (0,1]$, *we have*

$$\begin{aligned} & \bigl\vert I_{f,\eta }(\mu ,k;x,\lambda ,m,a,b) \bigr\vert \\ &\quad\leq C_{0}^{1-\frac{1}{q}}(k,\mu ,\lambda ) \\ &\quad\quad {} \times \biggl\{ \biggl\vert \frac{\eta ^{\frac{\mu }{k}+2}(x,a,m)}{(\frac{\mu }{k}+1)\eta (b,a,m)} \biggr\vert \bigl[ \bigl\vert f''(x) \bigr\vert ^{q}T_{1}(k, \mu ,\lambda ;s)+m \bigl\vert f''(a) \bigr\vert ^{q}T_{2}(k,\mu ,\lambda ;s) \bigr]^{\frac{1}{q}} \\ &\quad\quad {} + \biggl\vert \frac{(-1)^{\frac{\mu }{k}+2}\eta ^{\frac{\mu }{k}+2}(x,b,m)}{(\frac{\mu }{k}+1)\eta (b,a,m)} \biggr\vert \bigl[ \bigl\vert f''(x) \bigr\vert ^{q}T_{1}(k, \mu ,\lambda ;s)+m \bigl\vert f''(b) \bigr\vert ^{q}T_{2}(k,\mu ,\lambda ;s) \bigr]^{\frac{1}{q}} \biggr\} , \end{aligned}$$

*where we use the fact that*

$$\begin{aligned}& T_{1}(k,\mu ,\lambda ;s)= \textstyle\begin{cases} \frac{\frac{2\mu }{k} [ (\frac{\mu }{k}+1 )\lambda ]^{1+\frac{k(s+2)}{\mu }}}{(s+2)(\frac{\mu }{k}+s+2)}-\frac{(\frac{\mu }{k}+1)\lambda }{s+2}+\frac{1}{\frac{\mu }{k}+s+2}, &0\leq \lambda \leq \frac{1}{\frac{\mu }{k}+1}, \\ \frac{(\frac{\mu }{k}+1)\lambda }{s+2}-\frac{1}{\frac{\mu }{k}+s+2}, &\frac{1}{\frac{\mu }{k}+1}< \lambda \leq 1, \end{cases}\displaystyle \\& T_{2}(k,\mu ,\lambda ;s)= \textstyle\begin{cases} \left [ \textstyle\begin{array}{@{}c@{}} \beta (\frac{\mu }{k}+2,s+1 )- (\frac{\mu }{k}+1 )\lambda \beta (2,s+1 )\\ {}+2 (\frac{\mu }{k}+1 )\lambda \beta ( [ (\frac{\mu }{k}+1 )\lambda ]^{\frac{k}{\mu }};2,s+1 )\\ {}-2\beta ( [ (\frac{\mu }{k}+1 )\lambda ]^{\frac{k}{\mu }};\frac{\mu }{k}+2,s+1 ) \end{array}\displaystyle \right ] , &0\leq \lambda \leq \frac{1}{\frac{\mu }{k}+1}, \\ (\frac{\mu }{k}+1 )\lambda \beta (2,s+1)-\beta (\frac{\mu }{k}+2,s+1 ), &\frac{1}{\frac{\mu }{k}+1}< \lambda \leq 1, \end{cases}\displaystyle \end{aligned}$$

*and* $C_{0}(k,\mu ,\lambda )$ *is defined by* (2.5).

### Corollary 2.2

*In Theorem *2.1, *if the mapping* $\eta (b,a,m)=b-ma$ *along with* $m=1$, *taking* $x=\frac{a+b}{2}$, *then for* $s\in (0,1]$, *we have the following inequality for* *s*-*convex functions*:

$$\begin{aligned} & \biggl\vert \frac{2^{\frac{\mu }{k}-1}}{(b-a)^{\frac{\mu }{k}-1}}I_{f} \biggl(\mu ,k;\frac{a+b}{2},\lambda ,1,a,b \biggr) \biggr\vert \\ &\quad = \biggl\vert (1-\lambda )f \biggl(\frac{a+b}{2} \biggr)+\lambda \biggl[ \frac{f(a)+f(b)}{2} \biggr] \\ &\quad \quad {} -\frac{2^{\frac{\mu }{k}-1}\Gamma _{k}(\mu +k)}{(b-a)^{\frac{\mu }{k}}} \bigl[{}_{k}J^{\mu }_{(\frac{a+b}{2})^{-}} f(a)+{}_{k}J^{\mu }_{(\frac{a+b}{2})^{+}}f(b) \bigr] \biggr\vert \\ &\quad \leq \frac{(b-a)^{2}}{8 (\frac{\mu }{k}+1 )} C_{0}^{1-\frac{1}{q}}(k,\mu ,\lambda ) \biggl\{ \biggl[ \biggl\vert f'' \biggl(\frac{a+b}{2} \biggr) \biggr\vert ^{q}T_{1}(k,\mu ,\lambda ;s)+ \bigl\vert f''(a) \bigr\vert ^{q}T_{2}(k, \mu ,\lambda ;s) \biggr]^{\frac{1}{q}} \\ &\quad \quad {} + \biggl[ \biggl\vert f'' \biggl( \frac{a+b}{2} \biggr) \biggr\vert ^{q}T_{1}(k,\mu , \lambda ;s)+ \bigl\vert f''(b) \bigr\vert ^{q}T_{2}(k,\mu ,\lambda ;s) \biggr]^{\frac{1}{q}} \biggr\} . \end{aligned}$$

### Remark 2.2

In Corollary 2.2,

(i) taking $\lambda =\frac{1}{2}$, we obtain
  $$\begin{aligned} & \biggl\vert \frac{2^{\frac{\mu }{k}-1}}{(b-a)^{\frac{\mu }{k}-1}}I_{f} \biggl(\mu ,k;\frac{a+b}{2},\frac{1}{2},1,a,b \biggr) \biggr\vert \\ &\quad = \biggl\vert \frac{1}{4} \biggl[f(a)+2f \biggl(\frac{a+b}{2} \biggr)+f(b) \biggr]-\frac{2^{\frac{\mu }{k}-1}\Gamma _{k}(\mu +k)}{(b-a)^{\frac{\mu }{k}}} \bigl[{}_{k}J^{\mu }_{(\frac{a+b}{2})^{-}}f(a)+{}_{k}J^{\mu }_{(\frac{a+b}{2})^{+}}f(b) \bigr] \biggr\vert \\ &\quad \leq \frac{(b-a)^{2}}{8 (\frac{\mu }{k}+1 )} C_{0}^{1-\frac{1}{q}} \biggl(k,\mu , \frac{1}{2} \biggr) \\ &\quad \quad {} \times \biggl\{ \biggl[ \biggl\vert f'' \biggl(\frac{a+b}{2} \biggr) \biggr\vert ^{q}T_{1} \biggl(k,\mu ,\frac{1}{2};s \biggr)+ \bigl\vert f''(a) \bigr\vert ^{q}T_{2} \biggl(k,\mu ,\frac{1}{2};s \biggr) \biggr]^{\frac{1}{q}} \\ &\quad \quad {} + \biggl[ \biggl\vert f'' \biggl( \frac{a+b}{2} \biggr) \biggr\vert ^{q}T_{1} \biggl(k, \mu ,\frac{1}{2};s \biggr)+ \bigl\vert f''(b) \bigr\vert ^{q}T_{2} \biggl(k,\mu ,\frac{1}{2};s \biggr) \biggr]^{\frac{1}{q}} \biggr\} ; \end{aligned}$$
(ii) taking $\lambda =\frac{1}{3}$, we obtain
  $$\begin{aligned} & \biggl\vert \frac{2^{\frac{\mu }{k}-1}}{(b-a)^{\frac{\mu }{k}-1}}I_{f} \biggl(\mu ,k;\frac{a+b}{2},\frac{1}{3},1,a,b \biggr) \biggr\vert \\ &\quad = \biggl\vert \frac{1}{6} \biggl[f(a)+4f \biggl(\frac{a+b}{2} \biggr)+f(b) \biggr] \\ &\quad \quad {}-\frac{2^{\frac{\mu }{k}-1}\Gamma _{k}(\mu +k)}{(b-a)^{\frac{\mu }{k}}} \bigl[{}_{k}J^{\mu }_{(\frac{a+b}{2})^{-}}f(a)+{}_{k}J^{\mu }_{(\frac{a+b}{2})^{+}}f(b) \bigr] \biggr\vert \\ &\quad \leq \frac{(b-a)^{2}}{8 (\frac{\mu }{k}+1 )} C_{0}^{1-\frac{1}{q}} \biggl(k,\mu ,\frac{1}{3} \biggr) \\ &\quad \quad {} \times \biggl\{ \biggl[ \biggl\vert f'' \biggl(\frac{a+b}{2} \biggr) \biggr\vert ^{q}T_{1} \biggl(k,\mu ,\frac{1}{3};s \biggr)+ \bigl\vert f''(a) \bigr\vert ^{q}T_{2} \biggl(k,\mu ,\frac{1}{3};s \biggr) \biggr]^{\frac{1}{q}} \\ &\quad \quad {} + \biggl[ \biggl\vert f'' \biggl( \frac{a+b}{2} \biggr) \biggr\vert ^{q}T_{1} \biggl(k, \mu ,\frac{1}{3};s \biggr)+ \bigl\vert f''(b) \bigr\vert ^{q}T_{2} \biggl(k,\mu ,\frac{1}{3};s \biggr) \biggr]^{\frac{1}{q}} \biggr\} . \end{aligned}$$
  Specially, if we put $k=\mu =s=1$, then we obtain
  2.11 $$\begin{aligned} & \biggl\vert \frac{1}{6} \biggl[f(a)+4f \biggl(\frac{a+b}{2} \biggr)+f(b) \biggr]-\frac{1}{b-a} \int ^{b}_{a} f(x)\,\mathrm{d}x \biggr\vert \\ &\quad \leq \frac{(b-a)^{2}}{162} \biggl\{ \biggl[\frac{59}{96} \biggl\vert f'' \biggl(\frac{a+b}{2} \biggr) \biggr\vert ^{q}+\frac{37}{96} \bigl\vert f''(a) \bigr\vert ^{q} \biggr]^{\frac{1}{q}} \\ &\quad \quad {} + \biggl[\frac{59}{96} \biggl\vert f'' \biggl(\frac{a+b}{2} \biggr) \biggr\vert ^{q}+ \frac{37}{96} \bigl\vert f''(b) \bigr\vert ^{q} \biggr]^{\frac{1}{q}} \biggr\} \\ &\quad \leq \frac{(b-a)^{2}}{162} \biggl\{ \biggl(\frac{59\vert f''(a) \vert ^{q}+133\vert f''(b) \vert ^{q}}{192} \biggr)^{\frac{1}{q}} \\ &\quad \quad {} + \biggl(\frac{59\vert f''(b) \vert ^{q}+133\vert f''(a) \vert ^{q}}{192} \biggr)^{\frac{1}{q}} \biggr\} . \end{aligned}$$
  It is noted that the result of the first inequality in (2.11) is proved by İşcan in [34], which is better than the result presented by Sarikaya et al. in [36, Theorem 6].

### Remark 2.3

In Corollary 2.2, if we take $k=1$ and $\lambda =0,1$, we have the results (f) and (h) in [34, Corollary 2.3], respectively. Further, if we take $\mu =1$, we have the results (g) and (i) in [34, Corollary 2.3], respectively.

**II**. If $h(t)=t^{-s}$ in Theorem 2.1, then we have the following results.

### Corollary 2.3

*In Theorem *2.1, *if* $\vert f'' \vert ^{q}$ *for* $q\geq 1$ *is generalized* $(m,s)$-*Godunova–Levin–Dragomir*-*preinvex functions*, *then*, *for* $s\in (0,1)$ *and* $m\in (0,1]$, *we have*

$$\begin{aligned} & \bigl\vert I_{f,\eta }(\mu ,k;x,\lambda ,m,a,b) \bigr\vert \\ &\quad \leq C_{0}^{1-\frac{1}{q}}(k,\mu ,\lambda ) \\ &\quad \quad {} \times \biggl\{ \biggl\vert \frac{\eta ^{\frac{\mu }{k}+2}(x,a,m)}{(\frac{\mu }{k}+1)\eta (b,a,m)} \biggr\vert \bigl[ \bigl\vert f''(x) \bigr\vert ^{q}U_{1}(k, \mu ,\lambda ;s)+m \bigl\vert f''(a) \bigr\vert ^{q}U_{2}(k,\mu ,\lambda ;s) \bigr]^{\frac{1}{q}} \\ &\quad \quad {} + \biggl\vert \frac{(-1)^{\frac{\mu }{k}+2}\eta ^{\frac{\mu }{k}+2}(x,b,m)}{(\frac{\mu }{k}+1)\eta (b,a,m)} \biggr\vert \bigl[ \bigl\vert f''(x) \bigr\vert ^{q}U_{1}(k, \mu ,\lambda ;s)+m \bigl\vert f''(b) \bigr\vert ^{q}U_{2}(k,\mu ,\lambda ;s) \bigr]^{\frac{1}{q}} \biggr\} , \end{aligned}$$

*where we use the fact that*

$$\begin{aligned}& U_{1}(k,\mu ,\lambda ;s)= \textstyle\begin{cases} \frac{\frac{2\mu }{k} [ (\frac{\mu }{k}+1 )\lambda ]^{1+\frac{k(2-s)}{\mu }}}{(2-s)(\frac{\mu }{k}-s+2)}-\frac{(\frac{\mu }{k}+1)\lambda }{2-s}+\frac{1}{\frac{\mu }{k}-s+2}, &0\leq \lambda \leq \frac{1}{\frac{\mu }{k}+1}, \\ \frac{(\frac{\mu }{k}+1)\lambda }{2-s}-\frac{1}{\frac{\mu }{k}-s+2}, &\frac{1}{\frac{\mu }{k}+1}< \lambda \leq 1, \end{cases}\displaystyle \\& U_{2}(k,\mu ,\lambda ;s)= \textstyle\begin{cases} \left [ \textstyle\begin{array}{@{}c@{}} \beta (\frac{\mu }{k}+2,1-s )- (\frac{\mu }{k}+1 )\lambda \beta (2,1-s )\\ {}+2 (\frac{\mu }{k}+1 )\lambda \beta ( [ (\frac{\mu }{k}+1 )\lambda ]^{\frac{k}{\mu }};2,1-s ) \\ {}-2\beta ( [ (\frac{\mu }{k}+1 )\lambda ]^{\frac{k}{\mu }};\frac{\mu }{k}+2,1-s ) \end{array}\displaystyle \right ] , &0\leq \lambda \leq \frac{1}{\frac{\mu }{k}+1}, \\ (\frac{\mu }{k}+1 )\lambda \beta (2,1-s)-\beta (\frac{\mu }{k}+2,1-s ), &\frac{1}{\frac{\mu }{k}+1}< \lambda \leq 1, \end{cases}\displaystyle \end{aligned}$$

*and* $C_{0}(k,\mu ,\lambda )$ *is defined by* (2.5).

### Corollary 2.4

*In Theorem *2.1, *if the mapping* $\eta (b,a,m)=b-ma$ *together with* $m=1$, *taking* $x=\frac{a+b}{2}$, *for* $s\in (0,1)$, *we have the following inequality for* *s*-*Godunova–Levin functions*:

$$\begin{aligned} & \biggl\vert (1-\lambda )f \biggl(\frac{a+b}{2} \biggr)+\lambda \biggl[\frac{f(a)+f(b)}{2} \biggr] \\ &\quad \quad {} -\frac{2^{\frac{\mu }{k}-1}\Gamma _{k}(\mu +k)}{(b-a)^{\frac{\mu }{k}}} \bigl[{}_{k}J^{\mu }_{(\frac{a+b}{2})^{-}} f(a)+{}_{k}J^{\mu }_{(\frac{a+b}{2})^{+}}f(b) \bigr] \biggr\vert \\ &\quad \leq \frac{(b-a)^{2}}{8 (\frac{\mu }{k}+1 )} C_{0}^{1-\frac{1}{q}}(k,\mu ,\lambda ) \biggl\{ \biggl[ \biggl\vert f'' \biggl(\frac{a+b}{2} \biggr) \biggr\vert ^{q}U_{1}(k,\mu ,\lambda ;s)+ \bigl\vert f''(a) \bigr\vert ^{q}U_{2}(k, \mu ,\lambda ;s) \biggr]^{\frac{1}{q}} \\ &\quad \quad {} + \biggl[ \biggl\vert f'' \biggl( \frac{a+b}{2} \biggr) \biggr\vert ^{q}U_{1}(k,\mu , \lambda ;s)+ \bigl\vert f''(b) \bigr\vert ^{q}U_{2}(k,\mu ,\lambda ;s) \biggr]^{\frac{1}{q}} \biggr\} . \end{aligned}$$

### Remark 2.4

In Corollary 2.4,

- if $\lambda =\frac{1}{3}$, then we obtain
  $$\begin{aligned} & \biggl\vert \frac{2^{\frac{\mu }{k}-1}}{(b-a)^{\frac{\mu }{k}-1}}I_{f} \biggl(\mu ,k;\frac{a+b}{2},\frac{1}{3},1,a,b \biggr) \biggr\vert \\ &\quad = \biggl\vert \frac{1}{6} \biggl[f(a)+4f \biggl(\frac{a+b}{2} \biggr)+f(b) \biggr]-\frac{2^{\frac{\mu }{k}-1}\Gamma _{k}(\mu +k)}{(b-a)^{\frac{\mu }{k}}} \bigl[{}_{k}J^{\mu }_{(\frac{a+b}{2})^{-}} f(a)+{}_{k}J^{\mu }_{(\frac{a+b}{2})^{+}}f(b) \bigr] \biggr\vert \\ &\quad \leq \frac{(b-a)^{2}}{8 (\frac{\mu }{k}+1 )} C_{0}^{1-\frac{1}{q}} \biggl(k,\mu , \frac{1}{3} \biggr) \\ &\quad \quad {} \times \biggl\{ \biggl[ \biggl\vert f'' \biggl(\frac{a+b}{2} \biggr) \biggr\vert ^{q}U_{1} \biggl(k,\mu ,\frac{1}{3};s \biggr)+ \bigl\vert f''(a) \bigr\vert ^{q}U_{2} \biggl(k,\mu ,\frac{1}{3};s \biggr) \biggr]^{\frac{1}{q}} \\ &\quad \quad {} + \biggl[ \biggl\vert f'' \biggl( \frac{a+b}{2} \biggr) \biggr\vert ^{q}U_{1} \biggl(k, \mu ,\frac{1}{3};s \biggr)+ \bigl\vert f''(b) \bigr\vert ^{q}U_{2} \biggl(k,\mu ,\frac{1}{3};s \biggr) \biggr]^{\frac{1}{q}} \biggr\} . \end{aligned}$$
  Specially, if we put $k=1=\mu $, then we obtain a Simpson-type inequality:
  $$\begin{aligned} & \biggl\vert I_{f} \biggl(1,1; \frac{a+b}{2},\frac{1}{3},1,a,b \biggr) \biggr\vert \\ &\quad = \biggl\vert \frac{1}{6} \biggl[f(a)+4f \biggl(\frac{a+b}{2} \biggr)+f(b) \biggr]-\frac{1}{b-a} \int ^{b}_{a} f(x)\,\mathrm{d}x \biggr\vert \\ &\quad \leq \frac{(b-a)^{2}}{16} \biggl(\frac{8}{81} \biggr)^{1-\frac{1}{q}} \biggl\{ \biggl[ \biggl\vert f'' \biggl(\frac{a+b}{2}\biggr) \biggr\vert ^{q}U_{1} \biggl(1,1, \frac{1}{3};s \biggr) \\ &\quad \quad {}+ \bigl\vert f''(a) \bigr\vert ^{q}U_{2} \biggl(1,1,\frac{1}{3};s \biggr)\biggr]^{\frac{1}{q}} \\ &\quad \quad {} + \biggl[ \biggl\vert f'' \biggl(\frac{a+b}{2} \biggr) \biggr\vert ^{q}U_{1} \biggl(1,1,\frac{1}{3};s \biggr) + \bigl\vert f''(b)\bigr\vert ^{q}U_{2} \biggl(1,1,\frac{1}{3};s \biggr) \biggr]^{\frac{1}{q}} \biggr\} ; \end{aligned}$$
- if $\lambda =\frac{1}{2}$, then we obtain
  $$\begin{aligned} & \biggl\vert \frac{2^{\frac{\mu }{k}-1}}{(b-a)^{\frac{\mu }{k}-1}}I_{f} \biggl(\mu ,k;\frac{a+b}{2},\frac{1}{2},1,a,b \biggr) \biggr\vert \\ &\quad = \biggl\vert \frac{1}{4} \biggl[f(a)+2f \biggl(\frac{a+b}{2} \biggr)+f(b) \biggr]-\frac{2^{\frac{\mu }{k}-1}\Gamma _{k}(\mu +k)}{(b-a)^{\frac{\mu }{k}}} \bigl[{}_{k}J^{\mu }_{(\frac{a+b}{2})^{-}} f(a)+{}_{k}J^{\mu }_{(\frac{a+b}{2})^{+}}f(b) \bigr] \biggr\vert \\ &\quad \leq \frac{(b-a)^{2}}{8 (\frac{\mu }{k}+1 )} C_{0}^{1-\frac{1}{q}} \biggl(k,\mu , \frac{1}{2} \biggr) \\ &\quad \quad {} \times \biggl\{ \biggl[ \biggl\vert f'' \biggl(\frac{a+b}{2} \biggr) \biggr\vert ^{q}U_{1} \biggl(k,\mu ,\frac{1}{2};s \biggr)+ \bigl\vert f''(a) \bigr\vert ^{q}U_{2} \biggl(k,\mu ,\frac{1}{2};s \biggr) \biggr]^{\frac{1}{q}} \\ &\quad \quad {} + \biggl[ \biggl\vert f'' \biggl( \frac{a+b}{2} \biggr) \biggr\vert ^{q}U_{1} \biggl(k, \mu ,\frac{1}{2};s \biggr)+ \bigl\vert f''(b) \bigr\vert ^{q}U_{2} \biggl(k,\mu ,\frac{1}{2};s \biggr) \biggr]^{\frac{1}{q}} \biggr\} . \end{aligned}$$
  Specially, if we put $k=1=\mu $, then we obtain an averaged midpoint-trapezoid-type inequality:
  $$\begin{aligned} & \biggl\vert I_{f} \biggl(1,1; \frac{a+b}{2},\frac{1}{2},1,a,b \biggr) \biggr\vert \\ &\quad = \biggl\vert \frac{1}{4} \biggl[f(a)+2f \biggl(\frac{a+b}{2} \biggr)+f(b) \biggr]-\frac{1}{b-a} \int ^{b}_{a} f(x)\,\mathrm{d}x \biggr\vert \\ &\quad \leq \frac{(b-a)^{2}}{16} \biggl(\frac{1}{6} \biggr)^{1-\frac{1}{q}} \biggl\{ \biggl[ \biggl\vert f'' \biggl(\frac{a+b}{2} \biggr) \biggr\vert ^{q}U_{1} \biggl(1,1, \frac{1}{2};s \biggr)+ \bigl\vert f''(a) \bigr\vert ^{q}U_{2} \biggl(1,1,\frac{1}{2};s \biggr) \biggr]^{\frac{1}{q}} \\ &\quad \quad {} + \biggl[ \biggl\vert f'' \biggl( \frac{a+b}{2} \biggr) \biggr\vert ^{q}U_{1} \biggl(1,1,\frac{1}{2};s \biggr)+ \bigl\vert f''(b) \bigr\vert ^{q}U_{2} \biggl(1,1,\frac{1}{2};s \biggr) \biggr]^{\frac{1}{q}} \biggr\} ; \end{aligned}$$
- if $\lambda =0$, then we obtain
  $$\begin{aligned} & \biggl\vert \frac{2^{\frac{\mu }{k}-1}}{(b-a)^{\frac{\mu }{k}-1}}I_{f} \biggl(\mu ,k;\frac{a+b}{2},0,1,a,b \biggr) \biggr\vert \\ &\quad = \biggl\vert f \biggl(\frac{a+b}{2} \biggr)-\frac{2^{\frac{\mu }{k}-1}\Gamma _{k}(\mu +k)}{(b-a)^{\frac{\mu }{k}}} \bigl[{}_{k}J^{\mu }_{(\frac{a+b}{2})^{-}} f(a)+{}_{k}J^{\mu }_{(\frac{a+b}{2})^{+}}f(b) \bigr] \biggr\vert \\ &\quad \leq \frac{(b-a)^{2}}{8 (\frac{\mu }{k}+1 )} \biggl(\frac{1}{\frac{\mu }{k}+2} \biggr)^{1-\frac{1}{q}} \biggl\{ \biggl[\frac{\vert f''(\frac{a+b}{2}) \vert ^{q}}{\frac{\mu }{k}-s+2}+ \bigl\vert f''(a) \bigr\vert ^{q}\beta \biggl(\frac{\mu }{k}+2,1-s \biggr) \biggr]^{\frac{1}{q}} \\ &\quad \quad {} + \biggl[\frac{\vert f''(\frac{a+b}{2}) \vert ^{q}}{\frac{\mu }{k}-s+2}+ \bigl\vert f''(b) \bigr\vert ^{q}\beta \biggl(\frac{\mu }{k}+2,1-s \biggr) \biggr]^{\frac{1}{q}} \biggr\} . \end{aligned}$$
  Specially, if we put $k=1=\mu $, then we obtain a midpoint-type inequality:
  $$\begin{aligned} & \biggl\vert I_{f} \biggl(1,1; \frac{a+b}{2},0,1,a,b \biggr) \biggr\vert \\ &\quad = \biggl\vert f \biggl(\frac{a+b}{2} \biggr)-\frac{1}{b-a} \int ^{b}_{a} f(x)\,\mathrm{d}x \biggr\vert \\ &\quad \leq \frac{(b-a)^{2}}{16} \biggl(\frac{1}{3} \biggr)^{1-\frac{1}{q}} \biggl\{ \biggl[\frac{\vert f''(\frac{a+b}{2}) \vert ^{q}}{3-s}+ \bigl\vert f''(a) \bigr\vert ^{q}\beta (3,1-s ) \biggr]^{\frac{1}{q}} \\ &\quad \quad {}+ \biggl[\frac{\vert f''(\frac{a+b}{2}) \vert ^{q}}{3-s}+ \bigl\vert f''(b) \bigr\vert ^{q}\beta (3,1-s ) \biggr]^{\frac{1}{q}} \biggr\} ; \end{aligned}$$
- if $\lambda =1$, then we obtain
  $$\begin{aligned} & \biggl\vert \frac{2^{\frac{\mu }{k}-1}}{(b-a)^{\frac{\mu }{k}-1}}I_{f} \biggl(\mu ,k;\frac{a+b}{2},1,1,a,b \biggr) \biggr\vert \\ &\quad = \biggl\vert \frac{f(a)+f(b)}{2}-\frac{2^{\frac{\mu }{k}-1}\Gamma _{k}(\mu +k)}{(b-a)^{\frac{\mu }{k}}} \bigl[{}_{k}J^{\mu }_{(\frac{a+b}{2})^{-}} f(a)+{}_{k}J^{\mu }_{(\frac{a+b}{2})^{+}}f(b) \bigr] \biggr\vert \\ &\quad \leq \frac{(b-a)^{2}}{8 (\frac{\mu }{k}+1 )} \biggl[\frac{\frac{\mu }{k} (\frac{\mu }{k}+3 )}{2 (\frac{\mu }{k}+2 )} \biggr]^{1-\frac{1}{q}} \\ &\quad \quad {} \times \biggl\{ \biggl[\frac{\frac{\mu }{k} (\frac{\mu }{k}-s+3 )\vert f''(\frac{a+b}{2}) \vert ^{q}}{(2-s) (\frac{\mu }{k}-s+2 )} \\ &\quad \quad {} + \bigl\vert f''(a) \bigr\vert ^{q} \biggl( \biggl(\frac{\mu }{k}+1 \biggr)\beta (2,1-s)-\beta \biggl(\frac{\mu }{k}+2,1-s\biggr) \biggr) \biggr]^{\frac{1}{q}} \\ &\quad \quad {} + \biggl[\frac{\frac{\mu }{k} (\frac{\mu }{k}-s+3 )\vert f''(\frac{a+b}{2}) \vert ^{q}}{(2-s) (\frac{\mu }{k}-s+2 )} \\ &\quad \quad {} + \bigl\vert f''(b) \bigr\vert ^{q} \biggl( \biggl(\frac{\mu }{k}+1 \biggr)\beta (2,1-s)-\beta \biggl(\frac{\mu }{k}+2,1-s \biggr) \biggr) \biggr]^{\frac{1}{q}} \biggr\} . \end{aligned}$$
  Specially, if we put $k=1=\mu $, then we obtain a trapezoid-type inequality:
  $$\begin{aligned} & \biggl\vert I_{f} \biggl(1,1; \frac{a+b}{2},1,1,a,b \biggr) \biggr\vert \\ &\quad = \biggl\vert \frac{f(a)+f(b)}{2}-\frac{1}{b-a} \int ^{b}_{a} f(x)\,\mathrm{d}x \biggr\vert \\ &\quad \leq \frac{(b-a)^{2}}{16} \biggl(\frac{2}{3} \biggr)^{1-\frac{1}{q}} \biggl\{ \biggl[\frac{(4-s)\vert f''(\frac{a+b}{2}) \vert ^{q}}{(2-s)(3-s)} + \bigl\vert f''(a) \bigr\vert ^{q} \bigl(2\beta (2,1-s)-\beta (3,1-s) \bigr) \biggr]^{\frac{1}{q}} \\ &\quad \quad {} + \biggl[\frac{(4-s)\vert f''(\frac{a+b}{2}) \vert ^{q}}{(2-s)(3-s)} + \bigl\vert f''(b) \bigr\vert ^{q} \bigl(2\beta (2,1-s)-\beta (3,1-s) \bigr) \biggr]^{\frac{1}{q}} \biggr\} . \end{aligned}$$

**III**. If $h(t)=t(1-t)$ in Theorem 2.1, then we have the following results.

### Corollary 2.5

*In Theorem *2.1, *if* $\vert f'' \vert ^{q}$ *for* $q\geq 1$ *is generalized* $(m, tgs)$-*preinvex functions*, *then*, *for* $m\in (0,1]$, *we have*

$$ \begin{aligned} & \bigl\vert I_{f,\eta }(\mu ,k;x,\lambda ,m,a,b) \bigr\vert \\ &\quad \leq C_{0}^{1-\frac{1}{q}}(k,\mu ,\lambda )\theta ^{\frac{1}{q}}(k, \mu ,\lambda ) \biggl\{ \biggl\vert \frac{\eta ^{\frac{\mu }{k}+2}(x,a,m)}{(\frac{\mu }{k}+1)\eta (b,a,m)} \biggr\vert \bigl[ \bigl\vert f''(x) \bigr\vert ^{q}+m \bigl\vert f''(a) \bigr\vert ^{q} \bigr]^{\frac{1}{q}} \\ &\quad \quad {} + \biggl\vert \frac{(-1)^{\frac{\mu }{k}+2}\eta ^{\frac{\mu }{k}+2}(x,b,m)}{(\frac{\mu }{k}+1)\eta (b,a,m)} \biggr\vert \bigl[ \bigl\vert f''(x) \bigr\vert ^{q}+m \bigl\vert f''(b) \bigr\vert ^{q} \bigr]^{\frac{1}{q}} \biggr\} , \end{aligned} $$

*where we use the fact that*

$$ \theta (k,\mu ,\lambda )= \textstyle\begin{cases} \frac{\frac{2\mu }{k} [ (\frac{\mu }{k}+1 )\lambda ]^{\frac{3k}{\mu }+1}}{3(\frac{\mu }{k}+3)}-\frac{\frac{\mu }{k} [ (\frac{\mu }{k}+1 )\lambda ]^{\frac{4k}{\mu }+1}}{2(\frac{\mu }{k}+4)}-\frac{(\frac{\mu }{k}+1)\lambda }{12}+\frac{1}{(\frac{\mu }{k}+3)(\frac{\mu }{k}+4)}, &0\leq \lambda \leq \frac{1}{\frac{\mu }{k}+1}, \\ \frac{(\frac{\mu }{k}+1)\lambda }{12}-\frac{1}{(\frac{\mu }{k}+3)(\frac{\mu }{k}+4)}, &\frac{1}{\frac{\mu }{k}+1}< \lambda \leq 1\end{cases} $$

*and* $C_{0}(k,\mu ,\lambda )$ *is defined by* (2.5).

### Corollary 2.6

*In Theorem *2.1, *if the mapping* $\eta (b,a,m)=b-ma$ *along with* $m=1$, *taking* $x=\frac{a+b}{2}$, *we get the following inequality for* $tgs$-*convex functions*:

$$ \begin{aligned} & \biggl\vert (1-\lambda )f \biggl(\frac{a+b}{2} \biggr)+\lambda \biggl[\frac{f(a)+f(b)}{2} \biggr] \\ &\quad {} -\frac{2^{\frac{\mu }{k}-1}\Gamma _{k}(\mu +k)}{(b-a)^{\frac{\mu }{k}}} \bigl[{}_{k}J^{\mu }_{(\frac{a+b}{2})^{-}} f(a)+{}_{k}J^{\mu }_{(\frac{a+b}{2})^{+}}f(b) \bigr] \biggr\vert \\ &\leq \frac{(b-a)^{2}}{8 (\frac{\mu }{k}+1 )} C_{0}^{1-\frac{1}{q}}(k,\mu ,\lambda )\theta ^{\frac{1}{q}}(k,\mu ,\lambda ) \\ &\quad {} \times \biggl\{ \biggl[ \biggl\vert f'' \biggl(\frac{a+b}{2} \biggr) \biggr\vert ^{q}+ \bigl\vert f''(a) \bigr\vert ^{q} \biggr]^{\frac{1}{q}} + \biggl[ \biggl\vert f'' \biggl(\frac{a+b}{2} \biggr) \biggr\vert ^{q}+ \bigl\vert f''(b) \bigr\vert ^{q} \biggr]^{\frac{1}{q}} \biggr\} . \end{aligned} $$

### Remark 2.5

In Corollary 2.6,

- if $\lambda =\frac{1}{3}$, then we obtain
  $$\begin{aligned} & \biggl\vert \frac{1}{6} \biggl[f(a)+4f \biggl(\frac{a+b}{2} \biggr)+f(b) \biggr]-\frac{2^{\frac{\mu }{k}-1}\Gamma _{k}(\mu +k)}{(b-a)^{\frac{\mu }{k}}} \bigl[{}_{k}J^{\mu }_{(\frac{a+b}{2})^{-}} f(a)+{}_{k}J^{\mu }_{(\frac{a+b}{2})^{+}}f(b) \bigr] \biggr\vert \\ &\quad \leq \frac{(b-a)^{2}}{8 (\frac{\mu }{k}+1 )}C_{0}^{1-\frac{1}{q}} \biggl(k,\mu , \frac{1}{3} \biggr)\theta ^{\frac{1}{q}} \biggl(k,\mu ,\frac{1}{3} \biggr) \\ &\quad \quad {} \times \biggl\{ \biggl[ \biggl\vert f'' \biggl(\frac{a+b}{2} \biggr) \biggr\vert ^{q}+ \bigl\vert f''(a) \bigr\vert ^{q} \biggr]^{\frac{1}{q}} + \biggl[ \biggl\vert f'' \biggl(\frac{a+b}{2} \biggr) \biggr\vert ^{q}+ \bigl\vert f''(b) \bigr\vert ^{q} \biggr]^{\frac{1}{q}} \biggr\} . \end{aligned}$$
  Specially, if we put $k=1=\mu $, then we obtain a Simpson-type inequality:
  $$\begin{aligned} & \biggl\vert \frac{1}{6} \biggl[f(a)+4f \biggl(\frac{a+b}{2} \biggr)+f(b) \biggr]-\frac{1}{b-a} \int ^{b}_{a} f(x)\,\mathrm{d}x \biggr\vert \\ &\quad \leq \frac{(b-a)^{2}}{162}{ \biggl(\frac{23}{160} \biggr)}^{\frac{1}{q}} \\ &\quad \quad {} \times \biggl\{ \biggl[ \biggl\vert f'' \biggl(\frac{a+b}{2} \biggr) \biggr\vert ^{q}+ \bigl\vert f''(a) \bigr\vert ^{q} \biggr]^{\frac{1}{q}} + \biggl[ \biggl\vert f'' \biggl(\frac{a+b}{2} \biggr) \biggr\vert ^{q}+ \bigl\vert f''(b) \bigr\vert ^{q} \biggr]^{\frac{1}{q}} \biggr\} ; \end{aligned}$$
- if $\lambda =\frac{1}{2}$, then we obtain
  $$ \begin{aligned} & \biggl\vert \frac{1}{4} \biggl[f(a)+2f \biggl(\frac{a+b}{2} \biggr)+f(b) \biggr]-\frac{2^{\frac{\mu }{k}-1}\Gamma _{k}(\mu +k)}{(b-a)^{\frac{\mu }{k}}} \bigl[{}_{k}J^{\mu }_{(\frac{a+b}{2})^{-}}f(a)+{}_{k}J^{\mu }_{(\frac{a+b}{2})^{+}}f(b) \bigr] \biggr\vert \\ &\quad \leq \frac{(b-a)^{2}}{8 (\frac{\mu }{k}+1 )}C_{0}^{1-\frac{1}{q}} \biggl(k,\mu , \frac{1}{2} \biggr)\theta ^{\frac{1}{q}} \biggl(k,\mu ,\frac{1}{2} \biggr) \\ &\quad \quad {} \times \biggl\{ \biggl[ \biggl\vert f'' \biggl(\frac{a+b}{2} \biggr) \biggr\vert ^{q}+ \bigl\vert f''(a) \bigr\vert ^{q} \biggr]^{\frac{1}{q}} + \biggl[ \biggl\vert f'' \biggl(\frac{a+b}{2} \biggr) \biggr\vert ^{q}+ \bigl\vert f''(b) \bigr\vert ^{q} \biggr]^{\frac{1}{q}} \biggr\} . \end{aligned} $$
  Specially, if we put $k=1=\mu $, then we derive an averaged midpoint-trapezoid-type inequality:
  $$\begin{aligned} & \biggl\vert \frac{1}{4} \biggl[f(a)+2f \biggl(\frac{a+b}{2} \biggr)+f(b) \biggr]-\frac{1}{b-a} \int ^{b}_{a} f(x)\,\mathrm{d}x \biggr\vert \\ &\quad \leq \frac{(b-a)^{2}}{96}{ \biggl(\frac{1}{5} \biggr)}^{\frac{1}{q}} \biggl\{ \biggl[ \biggl\vert f'' \biggl( \frac{a+b}{2} \biggr) \biggr\vert ^{q}+ \bigl\vert f''(a) \bigr\vert ^{q} \biggr]^{\frac{1}{q}} \\ &\quad \quad {}+ \biggl[ \biggl\vert f'' \biggl(\frac{a+b}{2} \biggr) \biggr\vert ^{q}+ \bigl\vert f''(b) \bigr\vert ^{q} \biggr]^{\frac{1}{q}} \biggr\} ; \end{aligned}$$
- if $\lambda =0$, then we obtain
  $$ \begin{aligned} & \biggl\vert f \biggl(\frac{a+b}{2} \biggr)- \frac{2^{\frac{\mu }{k}-1}\Gamma _{k}(\mu +k)}{(b-a)^{\frac{\mu }{k}}} \bigl[{}_{k}J^{\mu }_{(\frac{a+b}{2})^{-}}f(a)+{}_{k}J^{\mu }_{(\frac{a+b}{2})^{+}}f(b) \bigr] \biggr\vert \\ &\quad \leq \frac{(b-a)^{2}}{8 (\frac{\mu }{k}+1 )} \biggl(\frac{1}{\frac{\mu }{k}+2} \biggr)^{1-\frac{1}{q}} \biggl[\frac{1}{(\frac{\mu }{k}+3)(\frac{\mu }{k}+4)} \biggr]^{\frac{1}{q}} \\ &\quad \quad {} \times \biggl\{ \biggl[ \biggl\vert f'' \biggl(\frac{a+b}{2} \biggr) \biggr\vert ^{q}+ \bigl\vert f''(a) \bigr\vert ^{q} \biggr]^{\frac{1}{q}} + \biggl[ \biggl\vert f'' \biggl(\frac{a+b}{2} \biggr) \biggr\vert ^{q}+ \bigl\vert f''(b) \bigr\vert ^{q} \biggr]^{\frac{1}{q}} \biggr\} . \end{aligned} $$
  Specially, if we put $k=1=\mu $, then we derive a midpoint-type inequality:
  $$\begin{aligned} & \biggl\vert f \biggl(\frac{a+b}{2} \biggr)- \frac{1}{b-a} \int ^{b}_{a} f(x)\,\mathrm{d}x \biggr\vert \\ &\quad \leq \frac{(b-a)^{2}}{48} \biggl(\frac{3}{20} \biggr)^{\frac{1}{q}} \biggl\{ \biggl[ \biggl\vert f'' \biggl(\frac{a+b}{2} \biggr) \biggr\vert ^{q}+ \bigl\vert f''(a) \bigr\vert ^{q} \biggr]^{\frac{1}{q}} \\ &\quad \quad {}+ \biggl[ \biggl\vert f'' \biggl(\frac{a+b}{2} \biggr) \biggr\vert ^{q}+ \bigl\vert f''(b) \bigr\vert ^{q} \biggr]^{\frac{1}{q}} \biggr\} ; \end{aligned}$$
- if $\lambda =1$, then we obtain
  $$\begin{aligned} & \biggl\vert \frac{f(a)+f(b)}{2}-\frac{2^{\frac{\mu }{k}-1}\Gamma _{k}(\mu +k)}{(b-a)^{\frac{\mu }{k}}} \bigl[{}_{k}J^{\mu }_{(\frac{a+b}{2})^{-}}f(a)+{}_{k}J^{\mu }_{(\frac{a+b}{2})^{+}}f(b) \bigr] \biggr\vert \\ &\quad \leq \frac{(b-a)^{2}}{8 (\frac{\mu }{k}+1 )} \biggl[\frac{\frac{\mu }{k} (\frac{\mu }{k}+3 )}{2 (\frac{\mu }{k}+2 )} \biggr]^{1-\frac{1}{q}} \biggl[\frac{\frac{\mu }{k} (\frac{\mu ^{2}}{k^{2}}+\frac{8\mu }{k}+19 )}{12 (\frac{\mu }{k}+3 ) (\frac{\mu }{k}+4 )} \biggr]^{\frac{1}{q}} \\ &\quad \quad {} \times \biggl\{ \biggl[ \biggl\vert f'' \biggl(\frac{a+b}{2} \biggr) \biggr\vert ^{q}+ \bigl\vert f''(a) \bigr\vert ^{q} \biggr]^{\frac{1}{q}} + \biggl[ \biggl\vert f'' \biggl(\frac{a+b}{2} \biggr) \biggr\vert ^{q}+ \bigl\vert f''(b) \bigr\vert ^{q} \biggr]^{\frac{1}{q}} \biggr\} . \end{aligned}$$
  Specially, if we put $k=1=\mu $, then we obtain a trapezoid-type inequality:
  $$\begin{aligned} & \biggl\vert \frac{f(a)+f(b)}{2}-\frac{1}{b-a} \int ^{b}_{a} f(x)\,\mathrm{d}x \biggr\vert \\ &\quad \leq \frac{(b-a)^{2}}{24} \biggl(\frac{7}{40} \biggr)^{\frac{1}{q}} \biggl\{ \biggl[ \biggl\vert f'' \biggl(\frac{a+b}{2} \biggr) \biggr\vert ^{q}+ \bigl\vert f''(a) \bigr\vert ^{q} \biggr]^{\frac{1}{q}} \\ &\quad \quad {}+ \biggl[ \biggl\vert f'' \biggl(\frac{a+b}{2} \biggr) \biggr\vert ^{q}+ \bigl\vert f''(b) \bigr\vert ^{q} \biggr]^{\frac{1}{q}} \biggr\} . \end{aligned}$$

**IV**. If $h(t)=\frac{\sqrt{t}}{2\sqrt{1-t}}$ in Theorem 2.1, then we have the following results.

### Corollary 2.7

*In Theorem *2.1, *if* $\vert f'' \vert ^{q}$ *for* $q\geq 1$ *is generalized* *m*-*MT*-*preinvex functions*, *then*, *for* $m\in (0,1]$, *we have*

$$ \begin{aligned} & \bigl\vert I_{f,\eta }(\mu ,k;x,\lambda ,m,a,b) \bigr\vert \\ &\quad \leq C_{0}^{1-\frac{1}{q}}(k,\mu ,\lambda ) \biggl\{ \biggl\vert \frac{\eta ^{\frac{\mu }{k}+2}(x,a,m)}{(\frac{\mu }{k}+1)\eta (b,a,m)} \biggr\vert \bigl[ \bigl\vert f''(x) \bigr\vert ^{q}\Phi _{1}(k,\mu ,\lambda )+m \bigl\vert f''(a) \bigr\vert ^{q}\Phi _{2}(k,\mu ,\lambda ) \bigr]^{\frac{1}{q}} \\ &\quad \quad {} + \biggl\vert \frac{(-1)^{\frac{\mu }{k}+2}\eta ^{\frac{\mu }{k}+2}(x,b,m)}{(\frac{\mu }{k}+1)\eta (b,a,m)} \biggr\vert \bigl[ \bigl\vert f''(x) \bigr\vert ^{q}\Phi _{1}(k,\mu ,\lambda )+m \bigl\vert f''(b) \bigr\vert ^{q}\Phi _{2}(k,\mu ,\lambda ) \bigr]^{\frac{1}{q}} \biggr\} , \end{aligned} $$

*where we use the fact that*

$$\begin{aligned}& \Phi _{1}(k,\mu ,\lambda ) \\& \quad = \textstyle\begin{cases} \left [ \textstyle\begin{array}{@{}c@{}} \frac{1}{2}\beta (\frac{\mu }{k}+\frac{5}{2},\frac{1}{2} )-\frac{3\lambda \pi (\frac{\mu }{k}+1)}{16}+ (\frac{\mu }{k}+1 )\lambda \beta ( [ (\frac{\mu }{k}+1 )\lambda ]^{\frac{k}{\mu }};\frac{5}{2},\frac{1}{2} )\\ {}-\beta ( [ (\frac{\mu }{k}+1 )\lambda ]^{\frac{k}{\mu }};\frac{\mu }{k}+\frac{5}{2},\frac{1}{2} )\end{array}\displaystyle \right ] , &0\leq \lambda \leq \frac{1}{\frac{\mu }{k}+1}, \\ \frac{3\lambda \pi (\frac{\mu }{k}+1)}{16}-\frac{1}{2}\beta (\frac{\mu }{k}+\frac{5}{2},\frac{1}{2} ), &\frac{1}{\frac{\mu }{k}+1}< \lambda \leq 1, \end{cases}\displaystyle \\& \Phi _{2}(k,\mu ,\lambda ) \\& \quad = \textstyle\begin{cases} \left [ \textstyle\begin{array}{@{}c@{}} \frac{1}{2}\beta (\frac{\mu }{k}+\frac{3}{2},\frac{3}{2} )-\frac{\lambda \pi (\frac{\mu }{k}+1)}{16} + (\frac{\mu }{k}+1 )\lambda \beta ( [ (\frac{\mu }{k}+1 )\lambda ]^{\frac{k}{\mu }};\frac{3}{2},\frac{3}{2} )\\ {}-\beta ( [ (\frac{\mu }{k}+1 )\lambda ]^{\frac{k}{\mu }};\frac{\mu }{k}+\frac{3}{2},\frac{3}{2} ) \end{array}\displaystyle \right ] , &0\leq \lambda \leq \frac{1}{\frac{\mu }{k}+1}, \\ \frac{\lambda \pi (\frac{\mu }{k}+1)}{16}-\frac{1}{2}\beta (\frac{\mu }{k}+\frac{3}{2},\frac{3}{2} ), &\frac{1}{\frac{\mu }{k}+1}< \lambda \leq 1, \end{cases}\displaystyle \end{aligned}$$

*and* $C_{0}(k,\mu ,\lambda )$ *is defined by* (2.5).

### Corollary 2.8

*In Theorem *2.1, *if the mapping* $\eta (b,a,m)=b-ma$ *together with* $m=1$, *taking* $x=\frac{a+b}{2}$, *we get the following inequality for* *MT*-*convex functions*:

$$\begin{aligned} & \biggl\vert (1-\lambda )f \biggl(\frac{a+b}{2} \biggr)+\lambda \biggl[\frac{f(a)+f(b)}{2} \biggr] \\ &\quad \quad {} -\frac{2^{\frac{\mu }{k}-1}\Gamma _{k}(\mu +k)}{(b-a)^{\frac{\mu }{k}}} \bigl[{}_{k}J^{\mu }_{(\frac{a+b}{2})^{-}} f(a)+{}_{k}J^{\mu }_{(\frac{a+b}{2})^{+}}f(b) \bigr] \biggr\vert \\ &\quad \leq \frac{(b-a)^{2}}{8 (\frac{\mu }{k}+1 )} C_{0}^{1-\frac{1}{q}}(k,\mu ,\lambda ) \biggl\{ \biggl[ \biggl\vert f'' \biggl(\frac{a+b}{2} \biggr) \biggr\vert ^{q}\Phi _{1}(k,\mu ,\lambda )+ \bigl\vert f''(a) \bigr\vert ^{q}\Phi _{2}(k,\mu ,\lambda ) \biggr]^{\frac{1}{q}} \\ &\quad \quad {} + \biggl[ \biggl\vert f'' \biggl( \frac{a+b}{2} \biggr) \biggr\vert ^{q}\Phi _{1}(k,\mu ,\lambda )+ \bigl\vert f''(b) \bigr\vert ^{q}\Phi _{2}(k,\mu ,\lambda ) \biggr]^{\frac{1}{q}} \biggr\} . \end{aligned}$$

### Remark 2.6

In Corollary 2.8,

- if $\lambda =\frac{1}{3}$, then we obtain
  $$\begin{aligned} & \biggl\vert \frac{1}{6} \biggl[f(a)+4f \biggl(\frac{a+b}{2} \biggr)+f(b) \biggr]-\frac{2^{\frac{\mu }{k}-1}\Gamma _{k}(\mu +k)}{(b-a)^{\frac{\mu }{k}}} \bigl[{}_{k}J^{\mu }_{(\frac{a+b}{2})^{-}} f(a)+{}_{k}J^{\mu }_{(\frac{a+b}{2})^{+}}f(b) \bigr] \biggr\vert \\ &\quad \leq \frac{(b-a)^{2}}{8 (\frac{\mu }{k}+1 )}C_{0}^{1-\frac{1}{q}} \biggl(k,\mu ,\frac{1}{3} \biggr) \biggl\{ \biggl[ \biggl\vert f'' \biggl(\frac{a+b}{2} \biggr) \biggr\vert ^{q}\Phi _{1} \biggl(k,\mu ,\frac{1}{3} \biggr) \\ &\quad \quad {}+ \bigl\vert f''(a) \bigr\vert ^{q}\Phi _{2} \biggl(k,\mu ,\frac{1}{3} \biggr) \biggr]^{\frac{1}{q}} \\ &\quad \quad {} + \biggl[ \biggl\vert f'' \biggl( \frac{a+b}{2} \biggr) \biggr\vert ^{q}\Phi _{1} \biggl(k,\mu ,\frac{1}{3} \biggr)+ \bigl\vert f''(b) \bigr\vert ^{q}\Phi _{2} \biggl(k,\mu ,\frac{1}{3} \biggr) \biggr]^{\frac{1}{q}} \biggr\} . \end{aligned}$$
  Specially, if we put $k=1=\mu $, then we derive a Simpson-type inequality:
  $$ \begin{aligned} & \biggl\vert \frac{1}{6} \biggl[f(a)+4f \biggl(\frac{a+b}{2} \biggr)+f(b) \biggr]-\frac{1}{b-a} \int ^{b}_{a} f(x)\,\mathrm{d}x \biggr\vert \\ &\quad \leq \frac{(b-a)^{2}}{16} { \biggl(\frac{8}{81} \biggr)}^{1-\frac{1}{q}} \biggl\{ \biggl[ \biggl\vert f'' \biggl( \frac{a+b}{2} \biggr) \biggr\vert ^{q}\Phi _{1} \biggl(1,1,\frac{1}{3} \biggr)+ \bigl\vert f''(a) \bigr\vert ^{q}\Phi _{2} \biggl(1,1,\frac{1}{3} \biggr) \biggr]^{\frac{1}{q}} \\ &\quad \quad {} + \biggl[ \biggl\vert f'' \biggl( \frac{a+b}{2} \biggr) \biggr\vert ^{q}\Phi _{1} \biggl(1,1,\frac{1}{3} \biggr)+ \bigl\vert f''(b) \bigr\vert ^{q}\Phi _{2} \biggl(1,1,\frac{1}{3} \biggr) \biggr]^{\frac{1}{q}} \biggr\} , \end{aligned} $$
  where
  $$ \Phi _{1} \biggl(1,1,\frac{1}{3} \biggr)=\frac{71\sqrt{2}}{648}+\frac{1}{8}\arcsin {\frac{\sqrt{3}}{3}}- \frac{\pi }{32} $$
  and
  $$ \Phi _{2} \biggl(1,1,\frac{1}{3} \biggr)=\frac{19\sqrt{2}}{648}+\frac{1}{12}\arcsin {\frac{1}{3}}+ \frac{1}{16}\arctan {2\sqrt{2}}-\frac{\pi }{32}; $$
- if $\lambda =\frac{1}{2}$, then we obtain
  $$\begin{aligned} & \biggl\vert \frac{1}{4} \biggl[f(a)+2f \biggl(\frac{a+b}{2} \biggr)+f(b) \biggr]-\frac{2^{\frac{\mu }{k}-1}\Gamma _{k}(\mu +k)}{(b-a)^{\frac{\mu }{k}}} \bigl[{}_{k}J^{\mu }_{(\frac{a+b}{2})^{-}} f(a)+{}_{k}J^{\mu }_{(\frac{a+b}{2})^{+}}f(b) \bigr] \biggr\vert \\ &\quad \leq \frac{(b-a)^{2}}{8 (\frac{\mu }{k}+1 )} C_{0}^{1-\frac{1}{q}} \biggl(k,\mu , \frac{1}{2} \biggr) \biggl\{ \biggl[ \biggl\vert f''\biggl(\frac{a+b}{2} \biggr) \biggr\vert ^{q}\Phi _{1} \biggl(k,\mu ,\frac{1}{2} \biggr) \\ &\quad \quad {}+ \bigl\vert f''(a) \bigr\vert ^{q}\Phi _{2} \biggl(k,\mu ,\frac{1}{2} \biggr) \biggr]^{\frac{1}{q}} \\ &\quad \quad {} + \biggl[ \biggl\vert f'' \biggl( \frac{a+b}{2} \biggr) \biggr\vert ^{q}\Phi _{1} \biggl(k,\mu ,\frac{1}{2} \biggr)+ \bigl\vert f''(b)\bigr\vert ^{q}\Phi _{2} \biggl(k,\mu ,\frac{1}{2} \biggr) \biggr]^{\frac{1}{q}} \biggr\} . \end{aligned}$$
  Specially, if we put $k=1=\mu $, then we derive an averaged midpoint-trapezoid-type inequality:
  $$\begin{aligned} & \biggl\vert \frac{1}{4} \biggl[f(a)+2f \biggl(\frac{a+b}{2} \biggr)+f(b) \biggr]-\frac{1}{b-a} \int ^{b}_{a} f(x)\,\mathrm{d}x \biggr\vert \\ &\quad \leq \frac{(b-a)^{2}}{96} \biggl(\frac{3\pi }{16} \biggr)^{\frac{1}{q}} \biggl\{ \biggl[ \biggl\vert f'' \biggl(\frac{a+b}{2} \biggr) \biggr\vert ^{q}+ \bigl\vert f''(a) \bigr\vert ^{q} \biggr]^{\frac{1}{q}} \\ &\quad \quad {}+ \biggl[ \biggl\vert f'' \biggl(\frac{a+b}{2} \biggr) \biggr\vert ^{q}+ \bigl\vert f''(b) \bigr\vert ^{q} \biggr]^{\frac{1}{q}} \biggr\} ; \end{aligned}$$
- if $\lambda =0$, then we obtain
  $$\begin{aligned} & \biggl\vert f \biggl(\frac{a+b}{2} \biggr)- \frac{2^{\frac{\mu }{k}-1}\Gamma _{k}(\mu +k)}{(b-a)^{\frac{\mu }{k}}} \bigl[{}_{k}J^{\mu }_{(\frac{a+b}{2})^{-}}f(a)+{}_{k}J^{\mu }_{(\frac{a+b}{2})^{+}}f(b) \bigr] \biggr\vert \\ &\quad \leq \frac{(b-a)^{2}}{8 (\frac{\mu }{k}+1 )} \biggl(\frac{1}{\frac{\mu }{k}+2} \biggr)^{1-\frac{1}{q}} \biggl(\frac{1}{2} \biggr)^{\frac{1}{q}} \\ &\quad \quad {} \times \biggl\{ \biggl[ \biggl\vert f'' \biggl(\frac{a+b}{2} \biggr) \biggr\vert ^{q}\beta \biggl( \frac{\mu }{k}+\frac{5}{2},\frac{1}{2} \biggr)+ \bigl\vert f''(a) \bigr\vert ^{q}\beta \biggl( \frac{\mu }{k}+\frac{3}{2},\frac{3}{2} \biggr) \biggr]^{\frac{1}{q}} \\ &\quad \quad {} + \biggl[ \biggl\vert f'' \biggl( \frac{a+b}{2} \biggr) \biggr\vert ^{q}\beta \biggl( \frac{\mu }{k}+\frac{5}{2},\frac{1}{2} \biggr)+ \bigl\vert f''(b) \bigr\vert ^{q}\beta \biggl( \frac{\mu }{k}+\frac{3}{2},\frac{3}{2} \biggr) \biggr]^{\frac{1}{q}} \biggr\} . \end{aligned}$$
  Specially, if we put $k=1=\mu $, then we obtain a midpoint-type inequality:
  $$\begin{aligned} & \biggl\vert f \biggl(\frac{a+b}{2} \biggr)- \frac{1}{b-a} \int ^{b}_{a} f(x)\,\mathrm{d}x \biggr\vert \\ &\quad \leq \frac{(b-a)^{2}}{48} \biggl(\frac{3}{2} \biggr)^{\frac{1}{q}} \\ &\quad\quad {} \times \biggl\{ \biggl[\frac{5\pi }{16} \biggl\vert f'' \biggl(\frac{a+b}{2} \biggr) \biggr\vert ^{q}+\frac{\pi }{16} \bigl\vert f''(a) \bigr\vert ^{q} \biggr]^{\frac{1}{q}} \\ &\quad \quad {}+ \biggl[\frac{5\pi }{16} \biggl\vert f'' \biggl(\frac{a+b}{2} \biggr) \biggr\vert ^{q}+\frac{\pi }{16} \bigl\vert f''(b) \bigr\vert ^{q} \biggr]^{\frac{1}{q}} \biggr\} ; \end{aligned}$$
- if $\lambda =1$, then we obtain
  $$\begin{aligned} & \biggl\vert \frac{f(a)+f(b)}{2}-\frac{2^{\frac{\mu }{k}-1}\Gamma _{k}(\mu +k)}{(b-a)^{\frac{\mu }{k}}} \bigl[{}_{k}J^{\mu }_{(\frac{a+b}{2})^{-}}f(a)+{}_{k}J^{\mu }_{(\frac{a+b}{2})^{+}}f(b) \bigr] \biggr\vert \\ &\quad \leq \frac{(b-a)^{2}}{8 (\frac{\mu }{k}+1 )} \biggl(\frac{\frac{\mu }{k}(\frac{\mu }{k}+3)}{2(\frac{\mu }{k}+2)} \biggr)^{1-\frac{1}{q}} \biggl\{ \biggl[ \biggl\vert f'' \biggl(\frac{a+b}{2} \biggr) \biggr\vert ^{q} \biggl(\frac{3(\frac{\mu }{k}+1)\pi }{16}-\frac{1}{2} \beta \biggl(\frac{\mu }{k}+\frac{5}{2},\frac{1}{2} \biggr) \biggr) \\ &\quad \quad {} + \bigl\vert f''(a) \bigr\vert ^{q} \biggl(\frac{(\frac{\mu }{k}+1)\pi }{16}-\frac{1}{2}\beta \biggl( \frac{\mu }{k}+\frac{3}{2},\frac{3}{2} \biggr) \biggr) \biggr]^{\frac{1}{q}} \\ &\quad \quad {} + \biggl[ \biggl\vert f'' \biggl( \frac{a+b}{2} \biggr) \biggr\vert ^{q} \biggl( \frac{3(\frac{\mu }{k}+1)\pi }{16}-\frac{1}{2}\beta \biggl(\frac{\mu }{k}+ \frac{5}{2},\frac{1}{2} \biggr) \biggr) \\ &\quad \quad {} + \bigl\vert f''(b) \bigr\vert ^{q} \biggl(\frac{(\frac{\mu }{k}+1)\pi }{16}-\frac{1}{2}\beta \biggl( \frac{\mu }{k}+\frac{3}{2},\frac{3}{2} \biggr) \biggr) \biggr]^{\frac{1}{q}} \biggr\} . \end{aligned}$$
  Specially, if we put $k=1=\mu $, then we obtain a trapezoid-type inequality:
  $$\begin{aligned} & \biggl\vert \frac{f(a)+f(b)}{2}-\frac{1}{b-a} \int ^{b}_{a} f(x)\,\mathrm{d}x \biggr\vert \\ &\quad \leq \frac{(b-a)^{2}}{16} \biggl(\frac{2}{3} \biggr)^{1-\frac{1}{q}} \\ &\quad \quad {} \times \biggl\{ \biggl[\frac{7\pi }{32} \biggl\vert f'' \biggl(\frac{a+b}{2} \biggr) \biggr\vert ^{q}+\frac{3\pi }{32} \bigl\vert f''(a) \bigr\vert ^{q} \biggr]^{\frac{1}{q}} \\ &\qquad {} + \biggl[\frac{7\pi }{32} \biggl\vert f'' \biggl(\frac{a+b}{2} \biggr) \biggr\vert ^{q}+\frac{3\pi }{32} \bigl\vert f''(b) \bigr\vert ^{q} \biggr]^{\frac{1}{q}} \biggr\} . \end{aligned}$$

Now, we get ready to state the second theorem as follows.

### Theorem 2.2

*Let* $A\subseteq \mathbb{R}$ *be an open* *m*-*invex subset with respect to* $\eta : A\times A\times (0,1] \rightarrow \mathbb{R}\setminus \{0\}$ *for some fixed* $m \in (0,1]$, *and let* $a, b \in A$, $a< b$ *with* $\eta (b,a,m)>0$. *Assume that* $f:A\rightarrow \mathbb{R}$ *is a twice differentiable function on* *A* *such that* $f''$ *is integrable on* $[ma,ma+\eta (b,a,m) ]$. *If* $\vert f'' \vert ^{q}$ *for* $q>1$ *is a generalized* $(m,h)$-*preinvex function with respect to* *η* *and* $h:[0,1]\rightarrow \mathbb{R}_{0}$, *then the following inequality for* *k*-*fractional integrals with* $x \in [a,b]$, $\lambda \in [0, 1]$, $\mu > 0$, $k>0$ *exists*:

2.12 $$\begin{aligned} & \bigl\vert I_{f,\eta }(\mu ,k;x,\lambda ,m,a,b) \bigr\vert \\ &\quad \leq C_{3}^{\frac{1}{p}}(k,\mu ,\lambda ,p) \\ &\quad \quad {} \times \biggl\{ \biggl\vert \frac{\eta ^{\frac{\mu }{k}+2}(x,a,m)}{(\frac{\mu }{k}+1)\eta (b,a,m)} \biggr\vert \biggl[ \bigl\vert f''(x) \bigr\vert ^{q} \int ^{1}_{0}h(t)\,\mathrm{d}t+m \bigl\vert f''(a) \bigr\vert ^{q} \int ^{1}_{0}h(1-t)\,\mathrm{d}t \biggr]^{\frac{1}{q}} \\ &\quad \quad {} + \biggl\vert \frac{(-1)^{\frac{\mu }{k}+2}\eta ^{\frac{\mu }{k}+2}(x,b,m)}{(\frac{\mu }{k}+1)\eta (b,a,m)} \biggr\vert \\ &\quad \quad {}\times \biggl[ \bigl\vert f''(x) \bigr\vert ^{q} \int ^{1}_{0}h(t)\,\mathrm{d}t+m \bigl\vert f''(b) \bigr\vert ^{q} \int ^{1}_{0}h(1-t)\,\mathrm{d}t \biggr]^{\frac{1}{q}} \biggr\} , \end{aligned}$$

*where* $p=\frac{q}{q-1}$ *and*

$$\begin{aligned} &C_{3}(k,\mu ,\lambda ,p) \\ &\quad = \textstyle\begin{cases} \frac{1}{p(\frac{\mu }{k}+1)+1},& \lambda =0, \\ \left [ \textstyle\begin{array}{@{}c@{}} \frac{k [ (\frac{\mu }{k}+1 )\lambda ]^{\frac{k+kp(\frac{\mu }{k}+1)}{\mu }}}{\mu }\beta (\frac{k(1+p)}{\mu },1+p )\\ {}+\frac{k [1- (\frac{\mu }{k}+1 )\lambda ]^{p+1}}{\mu (p+1)}{}_{2}F_{1} (1-\frac{k(1+p)}{\mu },1;p+2;1- (\frac{\mu }{k}+1 )\lambda ) \end{array}\displaystyle \right ] ,&0< \lambda \leq \frac{1}{\frac{\mu }{k}+1}, \\ \frac{k [ (\frac{\mu }{k}+1 )\lambda ]^{\frac{k+kp(\frac{\mu }{k}+1)}{\mu }}}{\mu }\beta (\frac{1}{(\frac{\mu }{k}+1)\lambda };\frac{k(1+p)}{\mu },1+p ), & \frac{1}{\frac{\mu }{k}+1}< \lambda \leq 1. \end{cases}\displaystyle \end{aligned}$$

### Proof

Using Lemma 2.1 and Hölder’s inequality, we have

2.13 $$\begin{aligned} & \bigl\vert I_{f,\eta }(\mu ,k;x,\lambda ,m,a,b) \bigr\vert \\ &\quad \leq \biggl\vert \frac{\eta ^{\frac{\mu }{k}+2}(x,a,m)}{(\frac{\mu }{k}+1)\eta (b,a,m)} \biggr\vert \int ^{1}_{0}t \biggl\vert \biggl( \frac{\mu }{k}+1 \biggr)\lambda -t^{\frac{\mu }{k}} \biggr\vert \bigl\vert f'' \bigl(ma+t\eta (x,a,m) \bigr) \bigr\vert \,\mathrm{d}t \\ &\quad \quad {} + \biggl\vert \frac{(-1)^{\frac{\mu }{k}+2}\eta ^{\frac{\mu }{k}+2}(x,b,m)}{(\frac{\mu }{k}+1)\eta (b,a,m)} \biggr\vert \int ^{1}_{0}t \biggl\vert \biggl( \frac{\mu }{k}+1 \biggr)\lambda -t^{\frac{\mu }{k}} \biggr\vert \bigl\vert f'' \bigl(mb+t\eta (x,b,m) \bigr) \bigr\vert \,\mathrm{d}t \\ &\quad \leq \biggl\vert \frac{\eta ^{\frac{\mu }{k}+2}(x,a,m)}{(\frac{\mu }{k}+1)\eta (b,a,m)} \biggr\vert \biggl[ \int ^{1}_{0}t^{p} \biggl\vert \biggl( \frac{\mu }{k}+1 \biggr)\lambda -t^{\frac{\mu }{k}} \biggr\vert ^{p}\,\mathrm{d}t \biggr]^{\frac{1}{p}} \\ &\quad \quad {} \times \biggl[ \int ^{1}_{0} \bigl\vert f'' \bigl(ma+t\eta (x,a,m) \bigr) \bigr\vert ^{q}\,\mathrm{d}t \biggr]^{\frac{1}{q}} \\ &\quad \quad {} + \biggl\vert \frac{(-1)^{\frac{\mu }{k}+2}\eta ^{\frac{\mu }{k}+2}(x,b,m)}{(\frac{\mu }{k}+1)\eta (b,a,m)} \biggr\vert \biggl[ \int ^{1}_{0}t^{p} \biggl\vert \biggl( \frac{\mu }{k}+1 \biggr)\lambda -t^{\frac{\mu }{k}} \biggr\vert ^{p}\,\mathrm{d}t \biggr]^{\frac{1}{p}} \\ &\quad \quad {} \times \biggl[ \int ^{1}_{0} \bigl\vert f'' \bigl(mb+t\eta (x,b,m) \bigr) \bigr\vert ^{q}\,\mathrm{d}t \biggr]^{\frac{1}{q}}. \end{aligned}$$

Since $\vert f'' \vert ^{q}$ is generalized $(m,h)$-preinvex on $[ma,ma+\eta (b,a,m)]$, we get

2.14 $$\begin{aligned} \int ^{1}_{0} \bigl\vert f'' \bigl(ma+t\eta (x,a,m) \bigr) \bigr\vert ^{q}\,\mathrm{d}t &\leq \int ^{1}_{0} \bigl[h(t) \bigl\vert f''(x) \bigr\vert ^{q}+mh(1-t) \bigl\vert f''(a) \bigr\vert ^{q} \bigr]\,\mathrm{d}t \\ &= \bigl\vert f''(x) \bigr\vert ^{q} \int ^{1}_{0}h(t)\,\mathrm{d}t+m \bigl\vert f''(a) \bigr\vert ^{q} \int ^{1}_{0}h(1-t)\,\mathrm{d}t, \end{aligned}$$

2.15 $$\begin{aligned} \int ^{1}_{0} \bigl\vert f'' \bigl(mb+t\eta (x,b,m) \bigr) \bigr\vert ^{q}\,\mathrm{d}t &\leq \int ^{1}_{0} \bigl[h(t) \bigl\vert f''(x) \bigr\vert ^{q}+mh(1-t) \bigl\vert f''(b) \bigr\vert ^{q} \bigr]\,\mathrm{d}t \\ &= \bigl\vert f''(x) \bigr\vert ^{q} \int ^{1}_{0}h(t)\,\mathrm{d}t+m \bigl\vert f''(b) \bigr\vert ^{q} \int ^{1}_{0}h(1-t)\,\mathrm{d}t, \end{aligned}$$

and

2.16 $$\begin{aligned}& C_{3}(k,\mu ,\lambda ,p) \\& \quad = \int ^{1}_{0}t^{p} \biggl\vert \biggl( \frac{\mu }{k}+1 \biggr)\lambda -t^{\frac{\mu }{k}} \biggr\vert ^{p}\,\mathrm{d}t \\& \quad =\textstyle\begin{cases} \int ^{1}_{0}t^{(\frac{\mu }{k}+1)p}\,\mathrm{d}t,& \lambda =0,\\ \left [ \textstyle\begin{array}{cc} \int ^{[(\frac{\mu }{k}+1)\lambda ]^{\frac{k}{\mu }}}_{0}t^{p}[(\frac{\mu }{k}+1)\lambda -t^{\frac{\mu }{k}}]^{p}\,\mathrm{d}t \\ +\int ^{1}_{[(\frac{\mu }{k}+1)\lambda ]^{\frac{k}{\mu }}}t^{p}[t^{\frac{\mu }{k}}-(\frac{\mu }{k}+1)\lambda ]^{p}\,\mathrm{d}t \end{array}\displaystyle \right ] , &0< \lambda \leq \frac{1}{\frac{\mu }{k}+1}, \\ \int ^{1}_{0}t^{p}[(\frac{\mu }{k}+1)\lambda -t^{\frac{\mu }{k}}]^{p}\,\mathrm{d}t, &\frac{1}{\frac{\mu }{k}+1}< \lambda \leq 1 \end{cases}\displaystyle \\& \quad = \textstyle\begin{cases} \frac{1}{p(\frac{\mu }{k}+1)+1},& \lambda =0, \\ \left [ \textstyle\begin{array}{@{}c@{}} \frac{k [ (\frac{\mu }{k}+1 )\lambda ]^{\frac{k+kp(\frac{\mu }{k}+1)}{\mu }}}{\mu }\beta (\frac{k(1+p)}{\mu },1+p )\\ {}+\frac{k [1- (\frac{\mu }{k}+1 )\lambda ]^{p+1}}{\mu (p+1)}{}_{2}F_{1} (1-\frac{k(1+p)}{\mu },1;p+2;1- (\frac{\mu }{k}+1 )\lambda ) \end{array}\displaystyle \right ] ,&0< \lambda \leq \frac{1}{\frac{\mu }{k}+1}, \\ \frac{k [ (\frac{\mu }{k}+1 )\lambda ]^{\frac{k+kp(\frac{\mu }{k}+1)}{\mu }}}{\mu }\beta (\frac{1}{(\frac{\mu }{k}+1)\lambda };\frac{k(1+p)}{\mu },1+p ), &\frac{1}{\frac{\mu }{k}+1}< \lambda \leq 1. \end{cases}\displaystyle \end{aligned}$$

Hence, if we use (2.14)–(2.16) in (2.13), we can get the desired result. This completes the proof. □

Let us point out some special cases of Theorem 2.2.

**I**. If $h(t)=t^{s}$ in Theorem 2.2, then we have the following results.

### Corollary 2.9

*In Theorem *2.2, *if we use the generalized* $(m,s)$-*Breckner*-*preinvexity of* $\vert f'' \vert ^{q}$ *along with* $q>1$ *and* $p=\frac{q}{q-1}$, *then*, *for* $s\in (0,1]$ *and* $m\in (0,1]$, *we have the following inequality*:

$$ \begin{aligned} & \bigl\vert I_{f,\eta }(\mu ,k;x,\lambda ,m,a,b) \bigr\vert \\ &\quad \leq C_{3}^{\frac{1}{p}}(k,\mu ,\lambda ,p) \biggl\{ \biggl\vert \frac{\eta ^{\frac{\mu }{k}+2}(x,a,m)}{(\frac{\mu }{k}+1)\eta (b,a,m)} \biggr\vert \biggl[\frac{\vert f''(x) \vert ^{q}+m\vert f''(a) \vert ^{q}}{s+1} \biggr]^{\frac{1}{q}} \\ &\quad \quad {} + \biggl\vert \frac{(-1)^{\frac{\mu }{k}+2}\eta ^{\frac{\mu }{k}+2}(x,b,m)}{(\frac{\mu }{k}+1)\eta (b,a,m)} \biggr\vert \biggl[ \frac{\vert f''(x) \vert ^{q}+m\vert f''(b) \vert ^{q}}{s+1} \biggr]^{\frac{1}{q}} \biggr\} . \end{aligned} $$

### Corollary 2.10

*In Theorem *2.2, *if the mapping* $\eta (b,a,m)=b-ma$ *together with* $m=1$, *choosing* $x=\frac{a+b}{2}$, *for* $s\in (0,1]$, *we have the following inequality for* *s*-*convex functions*:

$$ \begin{aligned} & \biggl\vert \frac{2^{\frac{\mu }{k}-1}}{(b-a)^{\frac{\mu }{k}-1}}I_{f} \biggl(\mu ,k;\frac{a+b}{2},\lambda ,1,a,b \biggr) \biggr\vert \\ &\quad \leq \frac{(b-a)^{2}}{8 (\frac{\mu }{k}+1 )}C_{3}^{\frac{1}{p}}(k,\mu ,\lambda ,p) \biggl\{ \biggl[\frac{\vert f''(x) \vert ^{q}+\vert f''(a) \vert ^{q}}{s+1} \biggr]^{\frac{1}{q}} + \biggl[ \frac{\vert f''(x) \vert ^{q}+\vert f''(b) \vert ^{q}}{s+1} \biggr]^{\frac{1}{q}} \biggr\} . \end{aligned} $$

### Remark 2.7

In Corollary 2.10,

(i) if $\lambda =\frac{1}{2}$, then we obtain
  $$ \begin{aligned} & \biggl\vert \frac{2^{\frac{\mu }{k}-1}}{(b-a)^{\frac{\mu }{k}-1}}I_{f} \biggl(\mu ,k;\frac{a+b}{2},\frac{1}{2},1,a,b \biggr) \biggr\vert \\ &\quad \leq \frac{(b-a)^{2}}{8 (\frac{\mu }{k}+1 )}C_{3}^{\frac{1}{p}} \biggl(k,\mu , \frac{1}{2},p \biggr) \biggl\{ \biggl[\frac{\vert f''(x) \vert ^{q}+\vert f''(a) \vert ^{q}}{s+1} \biggr]^{\frac{1}{q}} + \biggl[\frac{\vert f''(x) \vert ^{q}+\vert f''(b) \vert ^{q}}{s+1} \biggr]^{\frac{1}{q}} \biggr\} ; \end{aligned} $$
(ii) if $k=1$ and $\lambda =\frac{1}{3},1$, then we have the results (c) and (f) of Corollary 2.3 in [34], respectively. Further, if we choose $\mu =1$, then we have the results (d) and (g) of Corollary 2.3 in [34], respectively.

**II**. If $h(t)=t^{-s}$ in Theorem 2.2, then we have the following results.

### Corollary 2.11

*In Theorem *2.2, *if we use the generalized* $(m,s)$-*Godunova–Levin–Dragomir*-*preinvexity of* $\vert f'' \vert ^{q}$ *along with* $q>1$ *and* $p=\frac{q}{q-1}$, *then*, *for* $s\in (0,1)$ *and* $m\in (0,1]$, *we have the following inequality*:

$$\begin{aligned} & \bigl\vert I_{f,\eta }(\mu ,k;x,\lambda ,m,a,b) \bigr\vert \\ &\quad \leq C_{3}^{\frac{1}{p}}(k,\mu ,\lambda ,p)\frac{1}{{(1-s)}^{\frac{1}{q}}} \biggl\{ \biggl\vert \frac{\eta ^{\frac{\mu }{k}+2}(x,a,m)}{(\frac{\mu }{k}+1)\eta (b,a,m)} \biggr\vert \bigl[ \bigl\vert f''(x) \bigr\vert ^{q}+m \bigl\vert f''(a) \bigr\vert ^{q} \bigr]^{\frac{1}{q}} \\ &\quad \quad {} + \biggl\vert \frac{(-1)^{\frac{\mu }{k}+2}\eta ^{\frac{\mu }{k}+2}(x,b,m)}{(\frac{\mu }{k}+1)\eta (b,a,m)} \biggr\vert \bigl[ \bigl\vert f''(x) \bigr\vert ^{q}+m \bigl\vert f''(b) \bigr\vert ^{q} \bigr]^{\frac{1}{q}} \biggr\} . \end{aligned}$$

### Corollary 2.12

*In Theorem *2.2, *if the mapping* $\eta (b,a,m)=b-ma$ *along with* $m=1$, *choosing* $x=\frac{a+b}{2}$, *for* $s\in (0,1)$, *we have the following inequality for* *s*-*Godunova–Levin functions*:

$$\begin{aligned} & \biggl\vert (1-\lambda )f \biggl(\frac{a+b}{2} \biggr)+\lambda \biggl[\frac{f(a)+f(b)}{2} \biggr]-\frac{2^{\frac{\mu }{k}-1}\Gamma _{k}(\mu +k)}{(b-a)^{\frac{\mu }{k}}} \bigl[{}_{k}J^{\mu }_{(\frac{a+b}{2})^{-}} f(a)+{}_{k}J^{\mu }_{(\frac{a+b}{2})^{+}}f(b) \bigr] \biggr\vert \\ &\quad \leq \frac{(b-a)^{2}}{8(\frac{\mu }{k}+1)(1-s)^{\frac{1}{q}}}C_{3}^{\frac{1}{p}}(k,\mu ,\lambda ,p) \\ &\quad \quad {} \times \biggl\{ \biggl[ \biggl\vert f'' \biggl(\frac{a+b}{2} \biggr) \biggr\vert ^{q}+ \bigl\vert f''(a) \bigr\vert ^{q} \biggr]^{\frac{1}{q}} + \biggl[ \biggl\vert f'' \biggl(\frac{a+b}{2} \biggr) \biggr\vert ^{q}+ \bigl\vert f''(b) \bigr\vert ^{q} \biggr]^{\frac{1}{q}} \biggr\} . \end{aligned}$$

### Remark 2.8

In Corollary 2.12,

- if $\lambda =\frac{1}{3}$, then we obtain
  $$\begin{aligned} & \biggl\vert \frac{1}{6} \biggl[f(a)+4f \biggl(\frac{a+b}{2} \biggr)+f(b) \biggr]-\frac{2^{\frac{\mu }{k}-1}\Gamma _{k}(\mu +k)}{(b-a)^{\frac{\mu }{k}}} \bigl[{}_{k}J^{\mu }_{(\frac{a+b}{2})^{-}}f(a)+{}_{k}J^{\mu }_{(\frac{a+b}{2})^{+}}f(b) \bigr] \biggr\vert \\ &\quad \leq \frac{(b-a)^{2}}{8(\frac{\mu }{k}+1)(1-s)^{\frac{1}{q}}}C_{3}^{\frac{1}{p}} \biggl(k,\mu , \frac{1}{3},p \biggr) \\ &\quad \quad {} \times \biggl\{ \biggl[ \biggl\vert f'' \biggl(\frac{a+b}{2} \biggr) \biggr\vert ^{q}+ \bigl\vert f''(a) \bigr\vert ^{q} \biggr]^{\frac{1}{q}} + \biggl[ \biggl\vert f'' \biggl(\frac{a+b}{2} \biggr) \biggr\vert ^{q}+ \bigl\vert f''(b) \bigr\vert ^{q} \biggr]^{\frac{1}{q}} \biggr\} . \end{aligned}$$
  Specially, if we put $k=1=\mu $, then we derive a Simpson-type inequality:
  $$\begin{aligned} & \biggl\vert \frac{1}{6} \biggl[f(a)+4f \biggl(\frac{a+b}{2} \biggr)+f(b) \biggr]-\frac{1}{b-a} \int ^{b}_{a} f(x)\,\mathrm{d}x \biggr\vert \\ &\quad \leq \frac{(b-a)^{2}}{16(1-s)^{\frac{1}{q}}}C_{3}^{\frac{1}{p}} \biggl(1,1, \frac{1}{3},p \biggr) \\ &\quad \quad {} \times \biggl\{ \biggl[ \biggl\vert f'' \biggl(\frac{a+b}{2} \biggr) \biggr\vert ^{q}+ \bigl\vert f''(a) \bigr\vert ^{q} \biggr]^{\frac{1}{q}} + \biggl[ \biggl\vert f'' \biggl(\frac{a+b}{2} \biggr) \biggr\vert ^{q}+ \bigl\vert f''(b) \bigr\vert ^{q} \biggr]^{\frac{1}{q}} \biggr\} ; \end{aligned}$$
- if $\lambda =\frac{1}{2}$, then we obtain
  $$\begin{aligned} & \biggl\vert \frac{1}{4} \biggl[f(a)+2f \biggl(\frac{a+b}{2} \biggr)+f(b) \biggr]-\frac{2^{\frac{\mu }{k}-1}\Gamma _{k}(\mu +k)}{(b-a)^{\frac{\mu }{k}}} \bigl[{}_{k}J^{\mu }_{(\frac{a+b}{2})^{-}}f(a)+{}_{k}J^{\mu }_{(\frac{a+b}{2})^{+}}f(b) \bigr] \biggr\vert \\ &\quad \leq \frac{(b-a)^{2}}{8(\frac{\mu }{k}+1)(1-s)^{\frac{1}{q}}}C_{3}^{\frac{1}{p}} \biggl(k,\mu , \frac{1}{2},p \biggr) \\ &\quad \quad {} \times \biggl\{ \biggl[ \biggl\vert f'' \biggl(\frac{a+b}{2} \biggr) \biggr\vert ^{q}+ \bigl\vert f''(a) \bigr\vert ^{q} \biggr]^{\frac{1}{q}} + \biggl[ \biggl\vert f'' \biggl(\frac{a+b}{2} \biggr) \biggr\vert ^{q}+ \bigl\vert f''(b) \bigr\vert ^{q} \biggr]^{\frac{1}{q}} \biggr\} . \end{aligned}$$
  Specially, if we put $k=1=\mu $, then we derive an averaged midpoint-trapezoid-type inequality:
  $$\begin{aligned} & \biggl\vert \frac{1}{4} \biggl[f(a)+2f \biggl(\frac{a+b}{2} \biggr)+f(b) \biggr]-\frac{1}{b-a} \int ^{b}_{a} f(x)\,\mathrm{d}x \biggr\vert \\ &\quad \leq \frac{(b-a)^{2}}{16(1-s)^{\frac{1}{q}}}{\beta ^{\frac{1}{p}}(1+p,1+p)} \\ &\quad \quad {} \times \biggl\{ \biggl[ \biggl\vert f'' \biggl(\frac{a+b}{2} \biggr) \biggr\vert ^{q}+ \bigl\vert f''(a) \bigr\vert ^{q} \biggr]^{\frac{1}{q}} + \biggl[ \biggl\vert f'' \biggl(\frac{a+b}{2} \biggr) \biggr\vert ^{q}+ \bigl\vert f''(b) \bigr\vert ^{q} \biggr]^{\frac{1}{q}} \biggr\} ; \end{aligned}$$
- if $\lambda =0$, then we obtain
  $$\begin{aligned} & \biggl\vert f \biggl(\frac{a+b}{2} \biggr)- \frac{2^{\frac{\mu }{k}-1}\Gamma _{k}(\mu +k)}{(b-a)^{\frac{\mu }{k}}} \bigl[{}_{k}J^{\mu }_{(\frac{a+b}{2})^{-}}f(a)+{}_{k}J^{\mu }_{(\frac{a+b}{2})^{+}}f(b) \bigr] \biggr\vert \\ &\quad \leq \frac{(b-a)^{2}}{8(\frac{\mu }{k}+1)(1-s)^{\frac{1}{q}}}{ \biggl[\frac{1}{p (\frac{\mu }{k}+1 )+1} \biggr]}^{\frac{1}{p}} \\ &\quad \quad {} \times \biggl\{ \biggl[ \biggl\vert f'' \biggl(\frac{a+b}{2} \biggr) \biggr\vert ^{q}+ \bigl\vert f''(a) \bigr\vert ^{q} \biggr]^{\frac{1}{q}} + \biggl[ \biggl\vert f'' \biggl(\frac{a+b}{2} \biggr) \biggr\vert ^{q}+ \bigl\vert f''(b) \bigr\vert ^{q} \biggr]^{\frac{1}{q}} \biggr\} . \end{aligned}$$
  Specially, if we put $k=1=\mu $, then we derive a midpoint-type inequality:
  $$\begin{aligned} & \biggl\vert f \biggl(\frac{a+b}{2} \biggr)- \frac{1}{b-a} \int ^{b}_{a} f(x)\,\mathrm{d}x \biggr\vert \\ &\quad \leq \frac{(b-a)^{2}}{16(1-s)^{\frac{1}{q}}}{ \biggl(\frac{1}{2p+1} \biggr)}^{\frac{1}{p}} \\ &\quad \quad {} \times \biggl\{ \biggl[ \biggl\vert f'' \biggl(\frac{a+b}{2} \biggr) \biggr\vert ^{q}+ \bigl\vert f''(a) \bigr\vert ^{q} \biggr]^{\frac{1}{q}} + \biggl[ \biggl\vert f'' \biggl(\frac{a+b}{2} \biggr) \biggr\vert ^{q}+ \bigl\vert f''(b) \bigr\vert ^{q} \biggr]^{\frac{1}{q}} \biggr\} ; \end{aligned}$$
- if $\lambda =1$, then we obtain
  $$\begin{aligned} & \biggl\vert \frac{f(a)+f(b)}{2}-\frac{2^{\frac{\mu }{k}-1}\Gamma _{k}(\mu +k)}{(b-a)^{\frac{\mu }{k}}} \bigl[{}_{k}J^{\mu }_{(\frac{a+b}{2})^{-}}f(a)+{}_{k}J^{\mu }_{(\frac{a+b}{2})^{+}}f(b) \bigr] \biggr\vert \\ &\quad \leq \frac{(b-a)^{2}}{8(\frac{\mu }{k}+1)(1-s)^{\frac{1}{q}}}C_{3}^{\frac{1}{p}}(k,\mu ,1,p) \\ &\quad \quad {} \times \biggl\{ \biggl[ \biggl\vert f'' \biggl(\frac{a+b}{2} \biggr) \biggr\vert ^{q}+ \bigl\vert f''(a) \bigr\vert ^{q} \biggr]^{\frac{1}{q}} + \biggl[ \biggl\vert f'' \biggl(\frac{a+b}{2} \biggr) \biggr\vert ^{q}+ \bigl\vert f''(b) \bigr\vert ^{q} \biggr]^{\frac{1}{q}} \biggr\} . \end{aligned}$$
  Specially, if we put $k=1=\mu $, then we derive a trapezoid-type inequality:
  $$\begin{aligned} & \biggl\vert \frac{f(a)+f(b)}{2}-\frac{1}{b-a} \int ^{b}_{a} f(x)\,\mathrm{d}x \biggr\vert \\ &\quad \leq \frac{(b-a)^{2}}{16(1-s)^{\frac{1}{q}}}{ \biggl[2^{1+2p}\beta \biggl(\frac{1}{2};1+p,1+p \biggr) \biggr]}^{\frac{1}{p}} \\ &\quad \quad {} \times \biggl\{ \biggl[ \biggl\vert f'' \biggl(\frac{a+b}{2} \biggr) \biggr\vert ^{q}+ \bigl\vert f''(a) \bigr\vert ^{q} \biggr]^{\frac{1}{q}} + \biggl[ \biggl\vert f'' \biggl(\frac{a+b}{2} \biggr) \biggr\vert ^{q}+ \bigl\vert f''(b) \bigr\vert ^{q} \biggr]^{\frac{1}{q}} \biggr\} . \end{aligned}$$

**III**. If $h(t)=t(1-t)$ in Theorem 2.2, then we have the following results.

### Corollary 2.13

*In Theorem *2.2, *if we use the generalized* $(m,tgs)$-*preinvexity of* $\vert f'' \vert ^{q}$ *along with* $q>1$ *and* $p=\frac{q}{q-1}$, *then*, *for* $m\in (0,1]$, *we have the following inequality*:

$$\begin{aligned} & \bigl\vert I_{f,\eta }(\mu ,k;x,\lambda ,m,a,b) \bigr\vert \\ &\quad \leq C_{3}^{\frac{1}{p}}(k,\mu ,\lambda ,p){ \biggl( \frac{1}{6} \biggr)}^{\frac{1}{q}} \biggl\{ \biggl\vert \frac{\eta ^{\frac{\mu }{k}+2}(x,a,m)}{(\frac{\mu }{k}+1)\eta (b,a,m)} \biggr\vert \bigl[ \bigl\vert f''(x) \bigr\vert ^{q}+m \bigl\vert f''(a) \bigr\vert ^{q} \bigr]^{\frac{1}{q}} \\ &\quad \quad {} + \biggl\vert \frac{(-1)^{\frac{\mu }{k}+2}\eta ^{\frac{\mu }{k}+2}(x,b,m)}{(\frac{\mu }{k}+1)\eta (b,a,m)} \biggr\vert \bigl[ \bigl\vert f''(x) \bigr\vert ^{q}+m \bigl\vert f''(b) \bigr\vert ^{q} \bigr]^{\frac{1}{q}} \biggr\} , \end{aligned}$$

*where* $C_{3}(k,\mu ,\lambda ,p)$ *is defined by* (2.16).

### Corollary 2.14

*In Theorem *2.2, *if the mapping* $\eta (b,a,m)=b-ma$ *together with* $m=1$, *choosing* $x=\frac{a+b}{2}$, *we get the following inequality for* $tgs$-*convex functions*:

$$\begin{aligned} & \biggl\vert (1-\lambda )f \biggl(\frac{a+b}{2} \biggr)+\lambda \biggl[\frac{f(a)+f(b)}{2} \biggr] -\frac{2^{\frac{\mu }{k}-1}\Gamma _{k}(\mu +k)}{(b-a)^{\frac{\mu }{k}}} \bigl[{}_{k}J^{\mu }_{(\frac{a+b}{2})^{-}} f(a)+{}_{k}J^{\mu }_{(\frac{a+b}{2})^{+}}f(b) \bigr] \biggr\vert \\ &\quad \leq \frac{(b-a)^{2}}{8(\frac{\mu }{k}+1)} \biggl(\frac{1}{6} \biggr)^{\frac{1}{q}}C_{3}^{\frac{1}{p}}(k, \mu ,\lambda ,p) \\ &\quad \quad {} \times \biggl\{ \biggl[ \biggl\vert f'' \biggl(\frac{a+b}{2} \biggr) \biggr\vert ^{q}+ \bigl\vert f''(a) \bigr\vert ^{q} \biggr]^{\frac{1}{q}} + \biggl[ \biggl\vert f'' \biggl(\frac{a+b}{2} \biggr) \biggr\vert ^{q}+ \bigl\vert f''(b) \bigr\vert ^{q} \biggr]^{\frac{1}{q}} \biggr\} . \end{aligned}$$

### Remark 2.9

In Corollary 2.14,

- if $\lambda =\frac{1}{3}$, then we obtain
  $$\begin{aligned} & \biggl\vert \frac{1}{6} \biggl[f(a)+4f \biggl(\frac{a+b}{2} \biggr)+f(b) \biggr]-\frac{2^{\frac{\mu }{k}-1}\Gamma _{k}(\mu +k)}{(b-a)^{\frac{\mu }{k}}} \bigl[{}_{k}J^{\mu }_{(\frac{a+b}{2})^{-}} f(a)+{}_{k}J^{\mu }_{(\frac{a+b}{2})^{+}}f(b) \bigr] \biggr\vert \\ &\quad \leq \frac{(b-a)^{2}}{8(\frac{\mu }{k}+1)} \biggl(\frac{1}{6} \biggr)^{\frac{1}{q}}C_{3}^{\frac{1}{p}} \biggl(k,\mu ,\frac{1}{3},p \biggr) \\ &\quad \quad {} \times \biggl\{ \biggl[ \biggl\vert f'' \biggl(\frac{a+b}{2} \biggr) \biggr\vert ^{q}+ \bigl\vert f''(a) \bigr\vert ^{q} \biggr]^{\frac{1}{q}} + \biggl[ \biggl\vert f'' \biggl(\frac{a+b}{2} \biggr) \biggr\vert ^{q}+ \bigl\vert f''(b) \bigr\vert ^{q} \biggr]^{\frac{1}{q}} \biggr\} . \end{aligned}$$
  Specially, if we put $k=1=\mu $, then we derive a Simpson-type inequality:
  $$\begin{aligned} & \biggl\vert \frac{1}{6} \biggl[f(a)+4f \biggl(\frac{a+b}{2} \biggr)+f(b) \biggr]-\frac{1}{b-a} \int ^{b}_{a} f(x)\,\mathrm{d}x \biggr\vert \\ &\quad \leq \frac{(b-a)^{2}}{16} \biggl(\frac{1}{6} \biggr)^{\frac{1}{q}}C_{3}^{\frac{1}{p}} \biggl(1,1, \frac{1}{3},p \biggr) \\ &\quad \quad {} \times \biggl\{ \biggl[ \biggl\vert f'' \biggl(\frac{a+b}{2} \biggr) \biggr\vert ^{q}+ \bigl\vert f''(a) \bigr\vert ^{q} \biggr]^{\frac{1}{q}} + \biggl[ \biggl\vert f'' \biggl(\frac{a+b}{2} \biggr) \biggr\vert ^{q}+ \bigl\vert f''(b) \bigr\vert ^{q} \biggr]^{\frac{1}{q}} \biggr\} ; \end{aligned}$$
- if $\lambda =\frac{1}{2}$, then we obtain
  $$ \begin{aligned} & \biggl\vert \frac{1}{4} \biggl[f(a)+2f \biggl(\frac{a+b}{2} \biggr)+f(b) \biggr]-\frac{2^{\frac{\mu }{k}-1}\Gamma _{k}(\mu +k)}{(b-a)^{\frac{\mu }{k}}} \bigl[{}_{k}J^{\mu }_{(\frac{a+b}{2})^{-}}f(a)+{}_{k}J^{\mu }_{(\frac{a+b}{2})^{+}}f(b) \bigr] \biggr\vert \\ &\quad \leq \frac{(b-a)^{2}}{8(\frac{\mu }{k}+1)} \biggl(\frac{1}{6} \biggr)^{\frac{1}{q}}C_{3}^{\frac{1}{p}} \biggl(k,\mu ,\frac{1}{2},p \biggr) \\ &\quad \quad {} \times \biggl\{ \biggl[ \biggl\vert f'' \biggl(\frac{a+b}{2} \biggr) \biggr\vert ^{q}+ \bigl\vert f''(a) \bigr\vert ^{q} \biggr]^{\frac{1}{q}} + \biggl[ \biggl\vert f'' \biggl(\frac{a+b}{2} \biggr) \biggr\vert ^{q}+ \bigl\vert f''(b) \bigr\vert ^{q} \biggr]^{\frac{1}{q}} \biggr\} . \end{aligned} $$
  Specially, if we put $k=1=\mu $, then we derive an averaged midpoint-trapezoid-type inequality:
  $$ \begin{aligned} & \biggl\vert \frac{1}{4} \biggl[f(a)+2f \biggl(\frac{a+b}{2} \biggr)+f(b) \biggr]-\frac{1}{b-a} \int ^{b}_{a} f(x)\,\mathrm{d}x \biggr\vert \\ &\quad \leq \frac{(b-a)^{2}}{16} \biggl(\frac{1}{6} \biggr)^{\frac{1}{q}}\beta ^{\frac{1}{p}}(1+p,1+p) \\ &\quad \quad {} \times \biggl\{ \biggl[ \biggl\vert f'' \biggl(\frac{a+b}{2} \biggr) \biggr\vert ^{q}+ \bigl\vert f''(a) \bigr\vert ^{q} \biggr]^{\frac{1}{q}} + \biggl[ \biggl\vert f'' \biggl(\frac{a+b}{2} \biggr) \biggr\vert ^{q}+ \bigl\vert f''(b) \bigr\vert ^{q} \biggr]^{\frac{1}{q}} \biggr\} ; \end{aligned} $$
- if $\lambda =0$, then we obtain
  $$\begin{aligned} & \biggl\vert f \biggl(\frac{a+b}{2} \biggr)- \frac{2^{\frac{\mu }{k}-1}\Gamma _{k}(\mu +k)}{(b-a)^{\frac{\mu }{k}}} \bigl[{}_{k}J^{\mu }_{(\frac{a+b}{2})^{-}}f(a)+{}_{k}J^{\mu }_{(\frac{a+b}{2})^{+}}f(b) \bigr] \biggr\vert \\ &\quad \leq \frac{(b-a)^{2}}{8(\frac{\mu }{k}+1)} \biggl(\frac{1}{6} \biggr)^{\frac{1}{q}}{ \biggl[\frac{1}{p (\frac{\mu }{k}+1 )+1} \biggr]}^{\frac{1}{p}} \\ &\quad \quad {} \times \biggl\{ \biggl[ \biggl\vert f'' \biggl(\frac{a+b}{2} \biggr) \biggr\vert ^{q}+ \bigl\vert f''(a) \bigr\vert ^{q} \biggr]^{\frac{1}{q}} + \biggl[ \biggl\vert f'' \biggl(\frac{a+b}{2} \biggr) \biggr\vert ^{q}+ \bigl\vert f''(b) \bigr\vert ^{q} \biggr]^{\frac{1}{q}} \biggr\} . \end{aligned}$$
  Specially, if we put $k=1=\mu $, then we derive a midpoint-type inequality:
  $$\begin{aligned} & \biggl\vert f \biggl(\frac{a+b}{2} \biggr)- \frac{1}{b-a} \int ^{b}_{a} f(x)\,\mathrm{d}x \biggr\vert \\ &\quad \leq \frac{(b-a)^{2}}{16} \biggl(\frac{1}{6} \biggr)^{\frac{1}{q}}{ \biggl(\frac{1}{2p+1} \biggr)}^{\frac{1}{p}} \\ &\quad \quad {} \times \biggl\{ \biggl[ \biggl\vert f'' \biggl(\frac{a+b}{2} \biggr) \biggr\vert ^{q}+ \bigl\vert f''(a) \bigr\vert ^{q} \biggr]^{\frac{1}{q}} + \biggl[ \biggl\vert f'' \biggl(\frac{a+b}{2} \biggr) \biggr\vert ^{q}+ \bigl\vert f''(b) \bigr\vert ^{q} \biggr]^{\frac{1}{q}} \biggr\} ; \end{aligned}$$
- if $\lambda =1$, then we obtain
  $$ \begin{aligned} & \biggl\vert \frac{f(a)+f(b)}{2}-\frac{2^{\frac{\mu }{k}-1}\Gamma _{k}(\mu +k)}{(b-a)^{\frac{\mu }{k}}} \bigl[{}_{k}J^{\mu }_{(\frac{a+b}{2})^{-}}f(a)+{}_{k}J^{\mu }_{(\frac{a+b}{2})^{+}}f(b) \bigr] \biggr\vert \\ &\quad \leq \frac{(b-a)^{2}}{8(\frac{\mu }{k}+1)} \biggl(\frac{1}{6} \biggr)^{\frac{1}{q}}C_{3}^{\frac{1}{p}}(k, \mu ,1,p) \\ &\quad \quad {} \times \biggl\{ \biggl[ \biggl\vert f'' \biggl(\frac{a+b}{2} \biggr) \biggr\vert ^{q}+ \bigl\vert f''(a) \bigr\vert ^{q} \biggr]^{\frac{1}{q}} + \biggl[ \biggl\vert f'' \biggl(\frac{a+b}{2} \biggr) \biggr\vert ^{q}+ \bigl\vert f''(b) \bigr\vert ^{q} \biggr]^{\frac{1}{q}} \biggr\} . \end{aligned} $$
  Specially, if we put $k=1=\mu $, then we derive a trapezoid-type inequality:
  $$ \begin{aligned} & \biggl\vert \frac{f(a)+f(b)}{2}-\frac{1}{b-a} \int ^{b}_{a} f(x)\,\mathrm{d}x \biggr\vert \\ &\quad \leq \frac{(b-a)^{2}}{16} \biggl(\frac{1}{6} \biggr)^{\frac{1}{q}}{ \biggl[2^{1+2p}\beta \biggl(\frac{1}{2};1+p,1+p \biggr) \biggr]}^{\frac{1}{p}} \\ &\quad \quad {} \times \biggl\{ \biggl[ \biggl\vert f'' \biggl(\frac{a+b}{2} \biggr) \biggr\vert ^{q}+ \bigl\vert f''(a) \bigr\vert ^{q} \biggr]^{\frac{1}{q}} + \biggl[ \biggl\vert f'' \biggl(\frac{a+b}{2} \biggr) \biggr\vert ^{q}+ \bigl\vert f''(b) \bigr\vert ^{q} \biggr]^{\frac{1}{q}} \biggr\} . \end{aligned} $$

**IV**. If $h(t)=\frac{\sqrt{t}}{2\sqrt{1-t}}$ in Theorem 2.2, then we have the following results.

### Corollary 2.15

*In Theorem *2.2, *if we use the generalized* *m*-*MT*-*preinvexity of* $\vert f'' \vert ^{q}$ *along with* $q>1$ *and* $p=\frac{q}{q-1}$, *then*, *for* $m\in (0,1]$, *we have the following inequality*:

$$ \begin{aligned} & \bigl\vert I_{f,\eta }(\mu ,k;x,\lambda ,m,a,b) \bigr\vert \\ &\quad \leq C_{3}^{\frac{1}{p}}(k,\mu ,\lambda ,p) \biggl( \frac{\pi }{4} \biggr)^{\frac{1}{q}} \biggl\{ \biggl\vert \frac{\eta ^{\frac{\mu }{k}+2}(x,a,m)}{(\frac{\mu }{k}+1)\eta (b,a,m)} \biggr\vert \bigl[ \bigl\vert f''(x) \bigr\vert ^{q}+m \bigl\vert f''(a) \bigr\vert ^{q} \bigr]^{\frac{1}{q}} \\ &\quad \quad {} + \biggl\vert \frac{(-1)^{\frac{\mu }{k}+2}\eta ^{\frac{\mu }{k}+2}(x,b,m)}{(\frac{\mu }{k}+1)\eta (b,a,m)} \biggr\vert \bigl[ \bigl\vert f''(x) \bigr\vert ^{q}+m \bigl\vert f''(b) \bigr\vert ^{q} \bigr]^{\frac{1}{q}} \biggr\} , \end{aligned} $$

*where we use the fact that*

$$ \begin{aligned} \int ^{1}_{0}\frac{\sqrt{t}}{2\sqrt{1-t}}\,\mathrm{d}t= \int ^{1}_{0}\frac{\sqrt{1-t}}{2\sqrt{t}}\,\mathrm{d}t= \frac{\pi }{4}. \end{aligned} $$

### Corollary 2.16

*In Theorem *2.2, *if the mapping* $\eta (b,a,m)=b-ma$ *together with* $m=1$, *choosing* $x=\frac{a+b}{2}$, *we get the following inequality for* *MT*-*convex functions*:

$$\begin{aligned} & \biggl\vert (1-\lambda )f \biggl(\frac{a+b}{2} \biggr)+\lambda \biggl[\frac{f(a)+f(b)}{2} \biggr] \\ &\quad \quad {} -\frac{2^{\frac{\mu }{k}-1}\Gamma _{k}(\mu +k)}{(b-a)^{\frac{\mu }{k}}} \bigl[{}_{k}J^{\mu }_{(\frac{a+b}{2})^{-}} f(a)+{}_{k}J^{\mu }_{(\frac{a+b}{2})^{+}}f(b) \bigr] \biggr\vert \\ &\quad \leq \frac{(b-a)^{2}}{8(\frac{\mu }{k}+1)} \biggl(\frac{\pi }{4} \biggr)^{\frac{1}{q}}C_{3}^{\frac{1}{p}}(k,\mu ,\lambda ,p) \\ &\quad \quad {} \times \biggl\{ \biggl[ \biggl\vert f'' \biggl(\frac{a+b}{2} \biggr) \biggr\vert ^{q}+ \bigl\vert f''(a) \bigr\vert ^{q} \biggr]^{\frac{1}{q}} + \biggl[ \biggl\vert f'' \biggl(\frac{a+b}{2} \biggr) \biggr\vert ^{q}+ \bigl\vert f''(b) \bigr\vert ^{q} \biggr]^{\frac{1}{q}} \biggr\} . \end{aligned}$$

### Remark 2.10

In Corollary 2.16,

- if $\lambda =\frac{1}{3}$, then we obtain
  $$\begin{aligned} & \biggl\vert \frac{1}{6} \biggl[f(a)+4f \biggl(\frac{a+b}{2} \biggr)+f(b) \biggr] \\ &\quad \quad {}-\frac{2^{\frac{\mu }{k}-1}\Gamma _{k}(\mu +k)}{(b-a)^{\frac{\mu }{k}}} \bigl[{}_{k}J^{\mu }_{(\frac{a+b}{2})^{-}}f(a)+{}_{k}J^{\mu }_{(\frac{a+b}{2})^{+}}f(b) \bigr] \biggr\vert \\ &\quad \leq \frac{(b-a)^{2}}{8(\frac{\mu }{k}+1)} \biggl(\frac{\pi }{4} \biggr)^{\frac{1}{q}}C_{3}^{\frac{1}{p}} \biggl(k,\mu ,\frac{1}{3},p \biggr) \\ &\quad \quad {} \times \biggl\{ \biggl[ \biggl\vert f'' \biggl(\frac{a+b}{2} \biggr) \biggr\vert ^{q}+ \bigl\vert f''(a) \bigr\vert ^{q} \biggr]^{\frac{1}{q}} + \biggl[ \biggl\vert f'' \biggl(\frac{a+b}{2} \biggr) \biggr\vert ^{q}+ \bigl\vert f''(b) \bigr\vert ^{q} \biggr]^{\frac{1}{q}} \biggr\} . \end{aligned}$$
  Specially, if we put $k=1=\mu $, then we derive a Simpson-type inequality:
  $$\begin{aligned} & \biggl\vert \frac{1}{6} \biggl[f(a)+4f \biggl(\frac{a+b}{2} \biggr)+f(b) \biggr]-\frac{1}{b-a} \int ^{b}_{a} f(x)\,\mathrm{d}x \biggr\vert \\ &\quad \leq \frac{(b-a)^{2}}{16} \biggl(\frac{\pi }{4} \biggr)^{\frac{1}{q}}C_{3}^{\frac{1}{p}} \biggl(1,1,\frac{1}{3},p \biggr) \\ &\quad \quad {} \times \biggl\{ \biggl[ \biggl\vert f'' \biggl( \frac{a+b}{2} \biggr) \biggr\vert ^{q}+ \bigl\vert f''(a) \bigr\vert ^{q} \biggr]^{\frac{1}{q}} + \biggl[ \biggl\vert f'' \biggl(\frac{a+b}{2} \biggr) \biggr\vert ^{q}+ \bigl\vert f''(b) \bigr\vert ^{q} \biggr]^{\frac{1}{q}}\biggr\} ; \end{aligned}$$
- if $\lambda =\frac{1}{2}$, then we obtain
  $$\begin{aligned} & \biggl\vert \frac{1}{4} \biggl[f(a)+2f \biggl(\frac{a+b}{2} \biggr)+f(b) \biggr] \\ &\quad \quad {}-\frac{2^{\frac{\mu }{k}-1}\Gamma _{k}(\mu +k)}{(b-a)^{\frac{\mu }{k}}} \bigl[{}_{k}J^{\mu }_{(\frac{a+b}{2})^{-}}f(a)+{}_{k}J^{\mu }_{(\frac{a+b}{2})^{+}}f(b) \bigr] \biggr\vert \\ &\quad \leq \frac{(b-a)^{2}}{8(\frac{\mu }{k}+1)} \biggl(\frac{\pi }{4} \biggr)^{\frac{1}{q}}C_{3}^{\frac{1}{p}} \biggl(k,\mu ,\frac{1}{2},p \biggr) \\ &\quad \quad {} \times \biggl\{ \biggl[ \biggl\vert f'' \biggl(\frac{a+b}{2} \biggr) \biggr\vert ^{q}+ \bigl\vert f''(a) \bigr\vert ^{q} \biggr]^{\frac{1}{q}} + \biggl[ \biggl\vert f'' \biggl(\frac{a+b}{2} \biggr) \biggr\vert ^{q}+ \bigl\vert f''(b) \bigr\vert ^{q} \biggr]^{\frac{1}{q}} \biggr\} . \end{aligned}$$
  Specially, if we put $k=1=\mu $, then we derive an averaged midpoint-trapezoid-type inequality:
  $$\begin{aligned} & \biggl\vert \frac{1}{4} \biggl[f(a)+2f \biggl(\frac{a+b}{2} \biggr)+f(b) \biggr]-\frac{1}{b-a} \int ^{b}_{a} f(x)\,\mathrm{d}x \biggr\vert \\ &\quad \leq \frac{(b-a)^{2}}{16} \biggl(\frac{\pi }{4} \biggr)^{\frac{1}{q}}\beta ^{\frac{1}{p}}(1+p,1+p) \\ &\quad \quad {} \times \biggl\{ \biggl[ \biggl\vert f'' \biggl(\frac{a+b}{2} \biggr) \biggr\vert ^{q}+ \bigl\vert f''(a) \bigr\vert ^{q} \biggr]^{\frac{1}{q}} + \biggl[ \biggl\vert f'' \biggl(\frac{a+b}{2} \biggr) \biggr\vert ^{q}+ \bigl\vert f''(b) \bigr\vert ^{q} \biggr]^{\frac{1}{q}} \biggr\} ; \end{aligned}$$
- if $\lambda =0$, then we obtain
  $$\begin{aligned} & \biggl\vert f \biggl(\frac{a+b}{2} \biggr)- \frac{2^{\frac{\mu }{k}-1}\Gamma _{k}(\mu +k)}{(b-a)^{\frac{\mu }{k}}} \bigl[{}_{k}J^{\mu }_{(\frac{a+b}{2})^{-}}f(a)+{}_{k}J^{\mu }_{(\frac{a+b}{2})^{+}}f(b) \bigr] \biggr\vert \\ &\quad \leq \frac{(b-a)^{2}}{8(\frac{\mu }{k}+1)} \biggl(\frac{\pi }{4} \biggr)^{\frac{1}{q}}{ \biggl[\frac{1}{p (\frac{\mu }{k}+1 )+1} \biggr]}^{\frac{1}{p}} \\ &\quad\quad {} \times \biggl\{ \biggl[ \biggl\vert f'' \biggl(\frac{a+b}{2} \biggr) \biggr\vert ^{q}+ \bigl\vert f''(a) \bigr\vert ^{q} \biggr]^{\frac{1}{q}} + \biggl[ \biggl\vert f'' \biggl(\frac{a+b}{2} \biggr) \biggr\vert ^{q}+ \bigl\vert f''(b) \bigr\vert ^{q} \biggr]^{\frac{1}{q}} \biggr\} . \end{aligned}$$
  Specially, if we put $k=1=\mu $, then we derive a midpoint-type inequality:
  $$ \begin{aligned} & \biggl\vert f \biggl(\frac{a+b}{2} \biggr)- \frac{1}{b-a} \int ^{b}_{a} f(x)\,\mathrm{d}x \biggr\vert \\ &\quad \leq \frac{(b-a)^{2}}{16} \biggl(\frac{\pi }{4} \biggr)^{\frac{1}{q}}{ \biggl(\frac{1}{2p+1} \biggr)}^{\frac{1}{p}} \\ &\quad \quad {} \times \biggl\{ \biggl[ \biggl\vert f'' \biggl(\frac{a+b}{2} \biggr) \biggr\vert ^{q}+ \bigl\vert f''(a) \bigr\vert ^{q} \biggr]^{\frac{1}{q}} + \biggl[ \biggl\vert f'' \biggl(\frac{a+b}{2} \biggr) \biggr\vert ^{q}+ \bigl\vert f''(b) \bigr\vert ^{q} \biggr]^{\frac{1}{q}} \biggr\} ; \end{aligned} $$
- if $\lambda =1$, then we obtain
  $$ \begin{aligned} & \biggl\vert \frac{f(a)+f(b)}{2}-\frac{2^{\frac{\mu }{k}-1}\Gamma _{k}(\mu +k)}{(b-a)^{\frac{\mu }{k}}} \bigl[{}_{k}J^{\mu }_{(\frac{a+b}{2})^{-}}f(a)+{}_{k}J^{\mu }_{(\frac{a+b}{2})^{+}}f(b) \bigr] \biggr\vert \\ &\quad \leq \frac{(b-a)^{2}}{8(\frac{\mu }{k}+1)} \biggl(\frac{\pi }{4} \biggr)^{\frac{1}{q}}C_{3}^{\frac{1}{p}}(k, \mu ,1,p) \\ &\quad \quad {} \times \biggl\{ \biggl[ \biggl\vert f'' \biggl(\frac{a+b}{2} \biggr) \biggr\vert ^{q}+ \bigl\vert f''(a) \bigr\vert ^{q} \biggr]^{\frac{1}{q}} + \biggl[ \biggl\vert f'' \biggl(\frac{a+b}{2} \biggr) \biggr\vert ^{q}+ \bigl\vert f''(b) \bigr\vert ^{q} \biggr]^{\frac{1}{q}} \biggr\} . \end{aligned} $$
  Specially, if we put $k=1=\mu $, then we derive a trapezoid-type inequality:
  $$\begin{aligned} & \biggl\vert \frac{f(a)+f(b)}{2}-\frac{1}{b-a} \int ^{b}_{a} f(x)\,\mathrm{d}x \biggr\vert \\ &\quad \leq \frac{(b-a)^{2}}{16} \biggl(\frac{\pi }{4} \biggr)^{\frac{1}{q}}{ \biggl[2^{1+2p}\beta \biggl(\frac{1}{2};1+p,1+p \biggr) \biggr]}^{\frac{1}{p}} \\ &\quad \quad {} \times \biggl\{ \biggl[ \biggl\vert f'' \biggl(\frac{a+b}{2} \biggr) \biggr\vert ^{q}+ \bigl\vert f''(a) \bigr\vert ^{q} \biggr]^{\frac{1}{q}} + \biggl[ \biggl\vert f'' \biggl(\frac{a+b}{2} \biggr) \biggr\vert ^{q}+ \bigl\vert f''(b) \bigr\vert ^{q} \biggr]^{\frac{1}{q}} \biggr\} . \end{aligned}$$
